# Role of water in the formation of macromolecular structures

**DOI:** 10.1007/s00249-016-1161-y

**Published:** 2016-07-25

**Authors:** Peter L. Privalov, Colyn Crane-Robinson

**Affiliations:** 1 Department of Biology, Johns Hopkins University, Baltimore, MD 21218 USA; 2 Biophysics Laboratories, School of Biology, University of Portsmouth, Portsmouth, PO1 2DT UK

**Keywords:** Water, Hydration, Tissues, Proteins, DNA

## Abstract

This review shows that water in biological systems is not just a passive liquid solvent but also a partner in the formation of the structure of proteins, nucleic acids and their complexes, thereby contributing to the stability and flexibility required for their proper function. Reciprocally, biological macromolecules affect the state of the water contacting them, so that it is only partly in the normal liquid state, being somewhat ordered when bound to macromolecules. While the compaction of globular proteins results from the reluctance of their hydrophobic groups to interact with water, the collagen superhelix is maintained by water forming a hydroxyproline-controlled frame around this coiled-coil macromolecule. As for DNA, its stability and rigidity are linked to water fixed by AT pairs in the minor groove: this leads to the enthalpic contribution of AT pairs exceeding that of GC pairs, but this is overbalanced by their greater entropy contribution, with the result that AT pairs melt at lower temperatures than GCs. Loss of this water drives transcription factor binding to the minor groove.

## Water

The phenomenon of life is most intimately associated with water: the presence of water is regarded as a key indicator of life: all living objects are indeed ~80 % water. The ubiquitous presence of water raises the question as to why it is so important for living species, i.e., for the objects made up of thousands of other components. Is it just an appropriate solvent for all these components, in particular for specific biological macromolecules, i.e., the proteins and nucleic acids, or is it just a general solvent?

Water is indeed an unusual liquid with unique properties that distinguish it qualitatively from “normal” liquids. For example, liquid water is characterized by a very high heat capacity, much in excess of that of other liquids. Furthermore, it has a very high dielectric constant, making it an excellent solvent for a wide variety of organic and inorganic compounds. Moreover, the acidity of any system is provided by the water and that makes it the ultimate participant in many biochemical reactions.

**Fig. 1:**
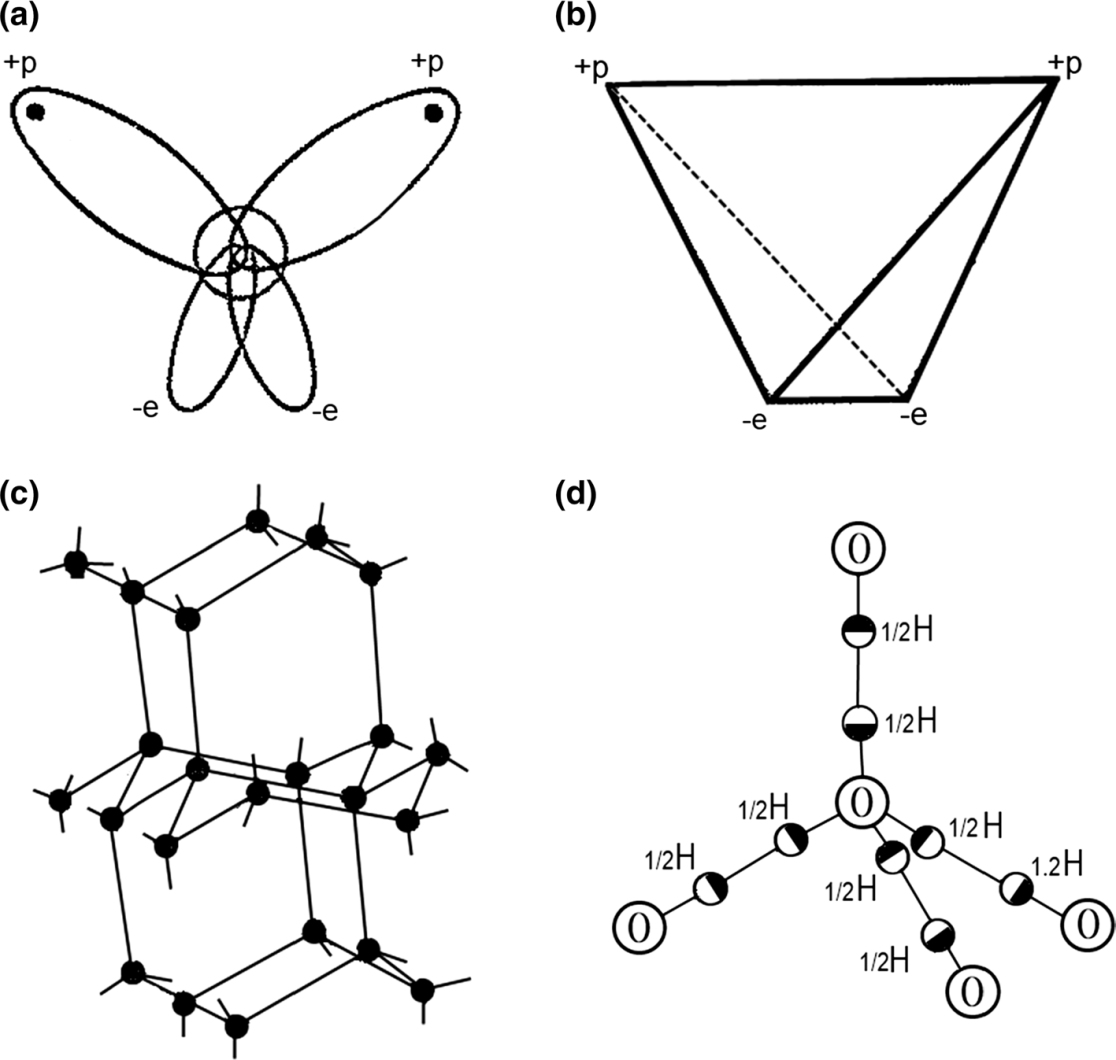
Distribution of charges in the water molecule (**a**) and its stereometry (**b**); **c** the structure of ice; **d** two possible positions for hydrogen localization in ice

The unusual properties of water are explained by the specific distribution of charges in this small molecule consisting of only one oxygen atom and two connected hydrogens (Fig. 1a, b). However, in the condensed state of water the hydrogens of each water molecule can form similar hydrogen bonds with the oxygens of neighboring water molecules (Fig. 1c). It is remarkable that the hydrogen atom in this very transparent lattice has two possible positions (Fig. 1d). Therefore, even at absolute zero temperature the ice crystal is not completely ordered, i.e., its entropy is not zero! Above 0 °C = 273.2 K, when the ice crystal structure breaks down, water still keeps its tendency to form crystal-like clusters. These clusters are unstable: they are “flickering” (Frank and Wen 1957). With temperature increase the probability of forming these flickering clusters decreases, so the order in water, which is due to these clusters, melts gradually, thereby providing the very substantial excess heat capacity to liquid water.

Correspondingly, water is classified as an *associative liquid*. The presence of various solutes could significantly affect the associative properties of water. So, the question is then: how liquid *is* water in biological systems, i.e., how ordered, or disordered, is it in comparison with pure water? This immediately raises the reciprocal question: how much does water affect the state and properties of biological macromolecules and their complexes?

## The bound water

### Water in biological tissues

The liquidity of water in biological objects, such as animal tissues, or aqueous solutions of biological macromolecules, can be judged by various physical criteria, of which the most practical and fundamental are the thermodynamic characteristics of water in these systems—which can be measured directly by calorimetry. For example, by measuring the heat effects associated with freezing and subsequent unfreezing of samples of tissues, one can determine how much water is freezable in these systems and thus judge how much water is unfreezable, i.e., is bound.

**Fig. 2:**
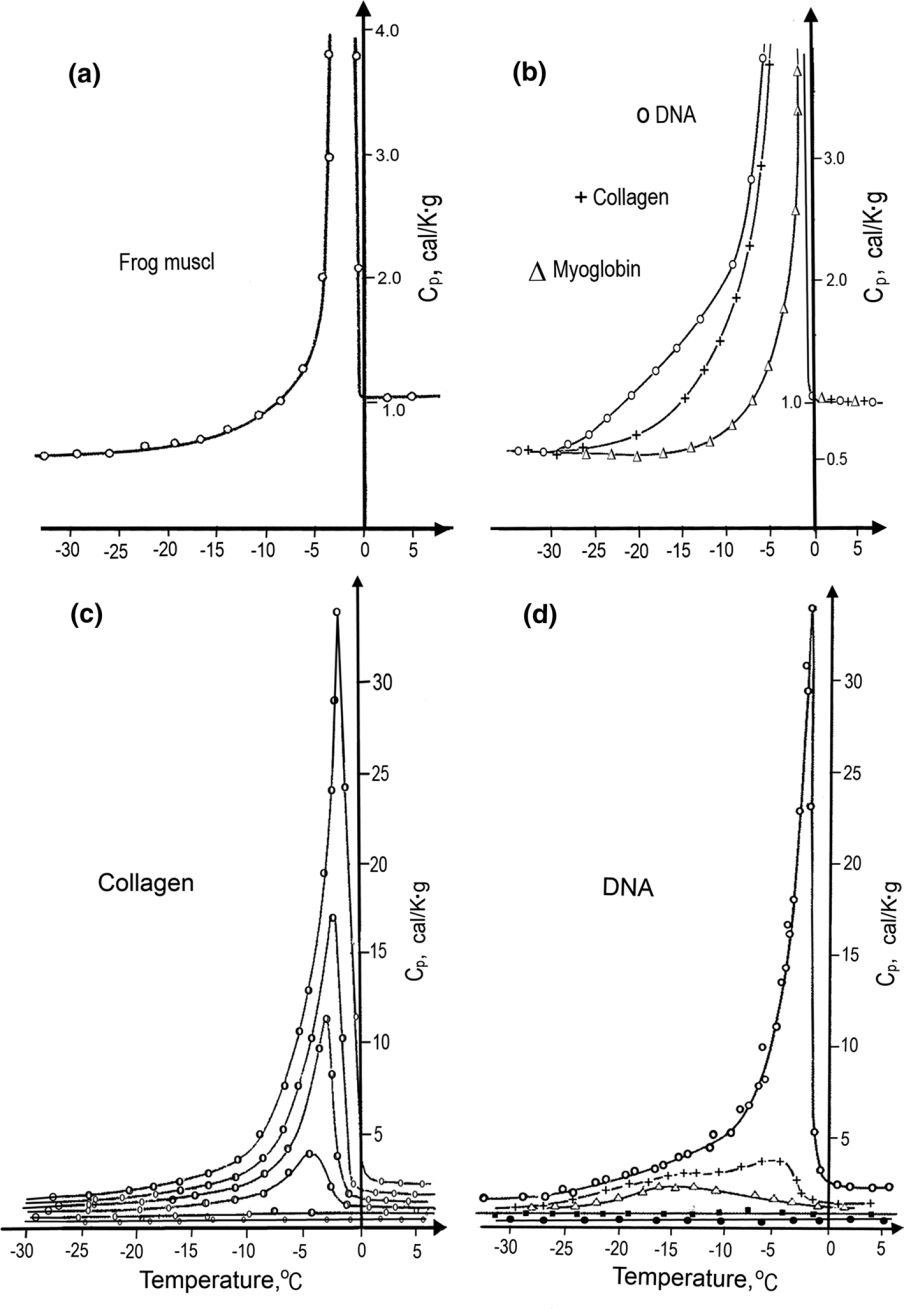
Temperature dependencies of the heat capacity of **a** frog muscle (Bella and Berman 1996); **b** solutions of DNA, collagen and hemoglobin containing 2 g of water per gram of biopolymer (Mrevlishvili and Privalov 1967); **c**, **d** collagen and DNA containing 0, 0.5, 0.75, 1.0 and 2.0 g of water per gram of macromolecule (Privalov and Mrevlishvili 1967)

The calorimetrically determined heat capacity profile of a frozen frog muscle shows that the excessive heat absorption associated with water unfreezing starts from about −25 °C and develops into a peak at 0 °C (Fig. 2a). However, the overall heat effect associated with the melting of water in this tissue appears smaller than expected for melting the whole amount of water present in this tissue, the amount of which is determined by vacuum drying the sample. Assuming that the enthalpy of water freezing is 6.00 kJ/mol (80 cal/g), one can define the “efficient” amount of unfreezable water: these are listed in Table 1 for various tissues. It appears that 1 g of frog muscle contains 0.25 g of unfreezable water; 1 g of liver contains 0.41 g of unfreezable water, while 1 g of rat brain contains only 0.15 g of unfreezable bound water. The last is understandable bearing in mined that brain contains large amounts of myelin, a highly inert insulator of nerves, which does not interact with water.

**Table 1:** The amount of bound (unfreezable) water in various tissues in grams per the gram of dry tissue/macromolecule (Mrevlishvili and Privalov 1967)

Sample	Unfreezable water (g/g)
Tissues	
Rat brain	0.15
Frog muscle	0.25
Frog liver	0.41
Silkworm eggs	0.38
Macromolecules	
DNA	0.610
Collagen	0.465
Serum albumin	0.315
Egg albumin	0.323
Myoglobin	0.324

### The aqueous solutions of biological macromolecules

One would a priori expect that the amount of bound water should be particularly large in the case of aqueous solutions of fibrilar macromolecules, such as collagen and DNA, which have larger relative exposed surfaces (i.e., the surface-to-mass ratio) than do compact globular proteins. Figure 2b shows the results of such investigations of the globular protein, myoglobin and the two fibrillar superhelices, collagen and DNA. These three samples have the same amount of water per gram of dry weight, but the melting profiles of water in the presence of these three macromolecules are very different. It appears also that the excess heat absorption upon heating the considered macromolecules in the presence of various amounts of water have rather complicated profiles (Fig. 2c, d): in the presence of less than 0.4 g of water per gram of collagen or per gram of DNA, their heating from −35 °C up to +5 °C does not reveal any excessive heat absorption around 0 °C, the temperature at which ordinary ice melts. Thus, all water present in these samples is tightly bound by the macromolecules. The excess heat effect appears only with higher water contents, and this excess heat absorption starts at temperatures significantly lower than 0 °C. Particularly, in the case of collagen it starts from about −25 °C and increases with increasing water content, while its maximum comes closer to 0 °C. In the case of DNA the excess heat absorption starts from −30 °C and proceeds in two distinct overlapping phases: one in the temperature range from −30 to −5 °C, the other in the range from −10 to 0 °C. It appears that the water, which is present above 0.5 g per gram of DNA, is still under the strong influence of the DNA, but this influence is of two different types, differing in the extent of the heat effect developed over two distinctly different temperature ranges.

From the deficit in the observed total enthalpy of water unfreezing, one can estimate the overall amount of water that does not participate in the freezing/unfreezing processes and can thus be supposed to be bound (Table 1). In the case of collagen, the amount of water that can be considered as bound appears to be about 0.5 g of water per gram of collagen. In the case of DNA it is larger, about 0.6 g of water per gram of DNA, while in the case of compact globular protein, myoglobin, it is much less, below 0.3 g of water per gram of protein. This is not surprising since the relative contact area with water of the rather compact globular protein is significantly lower than that of the fibrillar macromolecules. The question could, however, be inverted by asking why the globular proteins are compact, i.e., why do they have a relatively small surface area contacting water, while the DNA and collagen molecules have extended surfaces contacting water? Is this because most of the groups that form DNA or collagen want to be in contact with water, while most of the groups of globular proteins do not want to contact water? The *reverse* question is then: are proteins globular because water does not want to contact their polypeptide chains, while collagen and DNA are fibrillar because water wants to make contacts with their backbone groups? If so, *water does not appear just as a solvent for biological macromolecules, but also as a partner determining their structure and, therefore, their properties, being itself substantially influenced by these macromolecules.*

## Globular proteins in aqueous solution

### Heat denaturation of proteins

Globular proteins is assumed to mean compact, highly ordered proteins with molecular mass less than about 30 kDa. The polypeptide chain of globular proteins is tightly packed into a rather complicated unique conformation, which is determined by its primary structure, i.e., by the sequence of amino acid residues and thus by the sequence of the polar and apolar groups along the chain.

It is supposed that large proteins appeared in evolution as a result of association of small globular proteins or, more exactly, the genes coding the large globular proteins appeared as a result of association of the genes coding small ‘pro-proteins.’ These regions of large proteins still keep the folding pathways of their ancestors and form more or less independent domains in large proteins (for a review, see Privalov 1979).

Transfer of globular proteins to extreme conditions (e.g., high temperature, high pressure, high acidity, or high concentration of denaturants) leads to unfolding of their polypeptide chains, resulting in disappearance of all their unique biological functions, i.e., to their denaturation (for a review, see Mrevlishvili and Privalov 1967). Therefore, the study of protein denaturation is the only practical approach for understanding the mechanism of formation of their native structure, i.e., understanding the forces involved in this process and the cooperation of these forces in driving folding. Calorimetry plays a central role in these studies, providing direct information on the enthalpy and entropy of the process of protein unfolding/refolding (Privalov 1990).

**Fig. 3:**
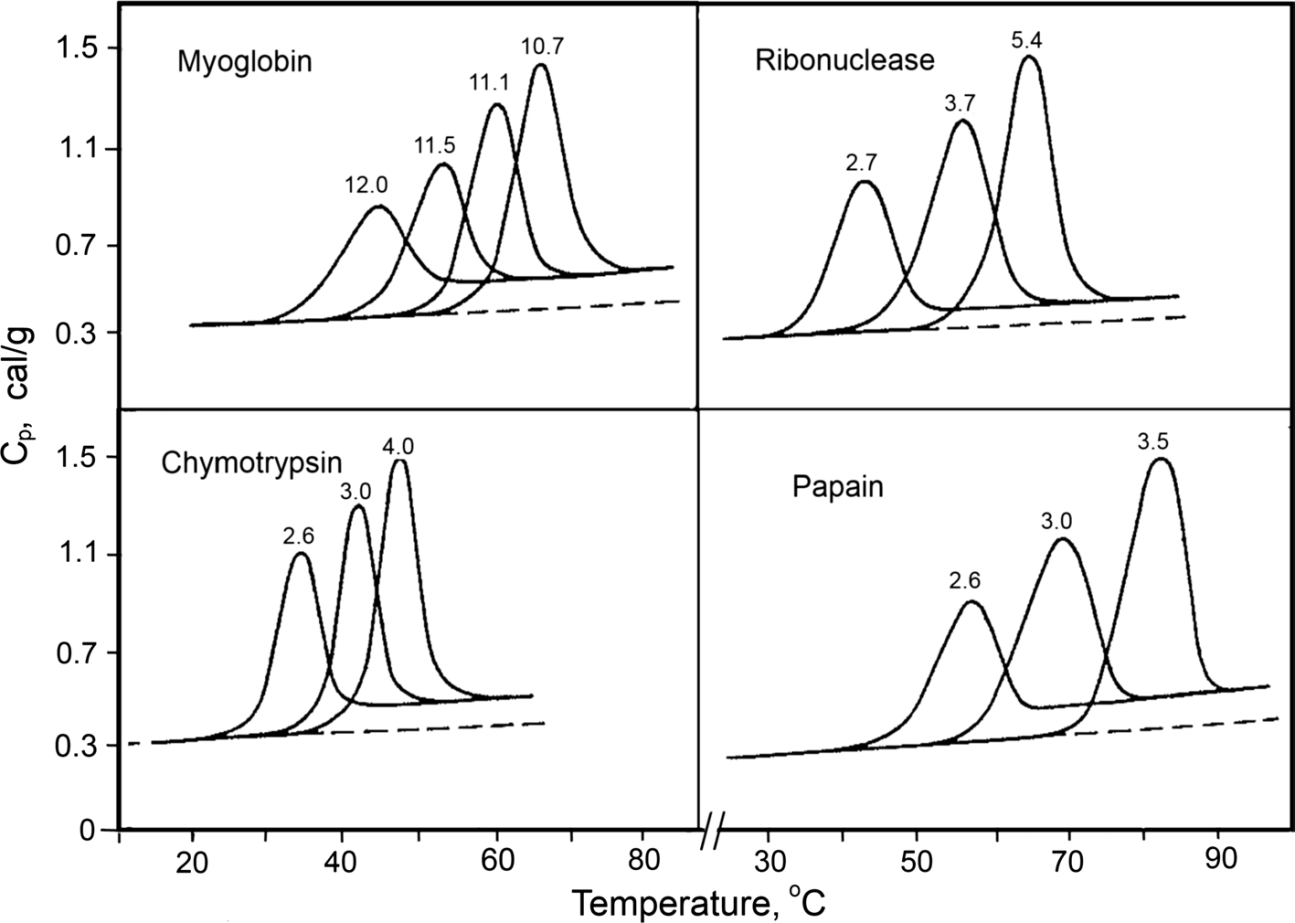
Partial specific heat capacity profiles of various globular proteins in solution having the indicated pH values (Privalov and Khechinashvili 1974)

The temperature-induced unfolding of globular proteins usually proceeds in a short temperature range with extensive heat absorption and results in a significant increase of their heat capacity (Fig. 3). The sharpness of the temperature-induced unfolding of globular proteins suggests that this is a highly cooperative process. It typically represents a two-state transition occurring without visible intermediates, i.e., all intermediates in protein folding/unfolding are highly unstable.

The significant heat capacity increment upon unfolding is a very specific feature of globular proteins, distinguishing them from other biological macromolecules. This heat capacity increment does not depend significantly on temperature, and its value is very specific for the given protein (Privalov and Makhatadze 1992). It turns out that its magnitude is proportional to the number of contacts between nonpolar groups in the protein and is thus determined by the overall surface area of the nonpolar groups that are exposed to water upon globular protein unfolding (Privalov 2012; Privalov and Khechinashvili 1974).

**Fig. 4:**
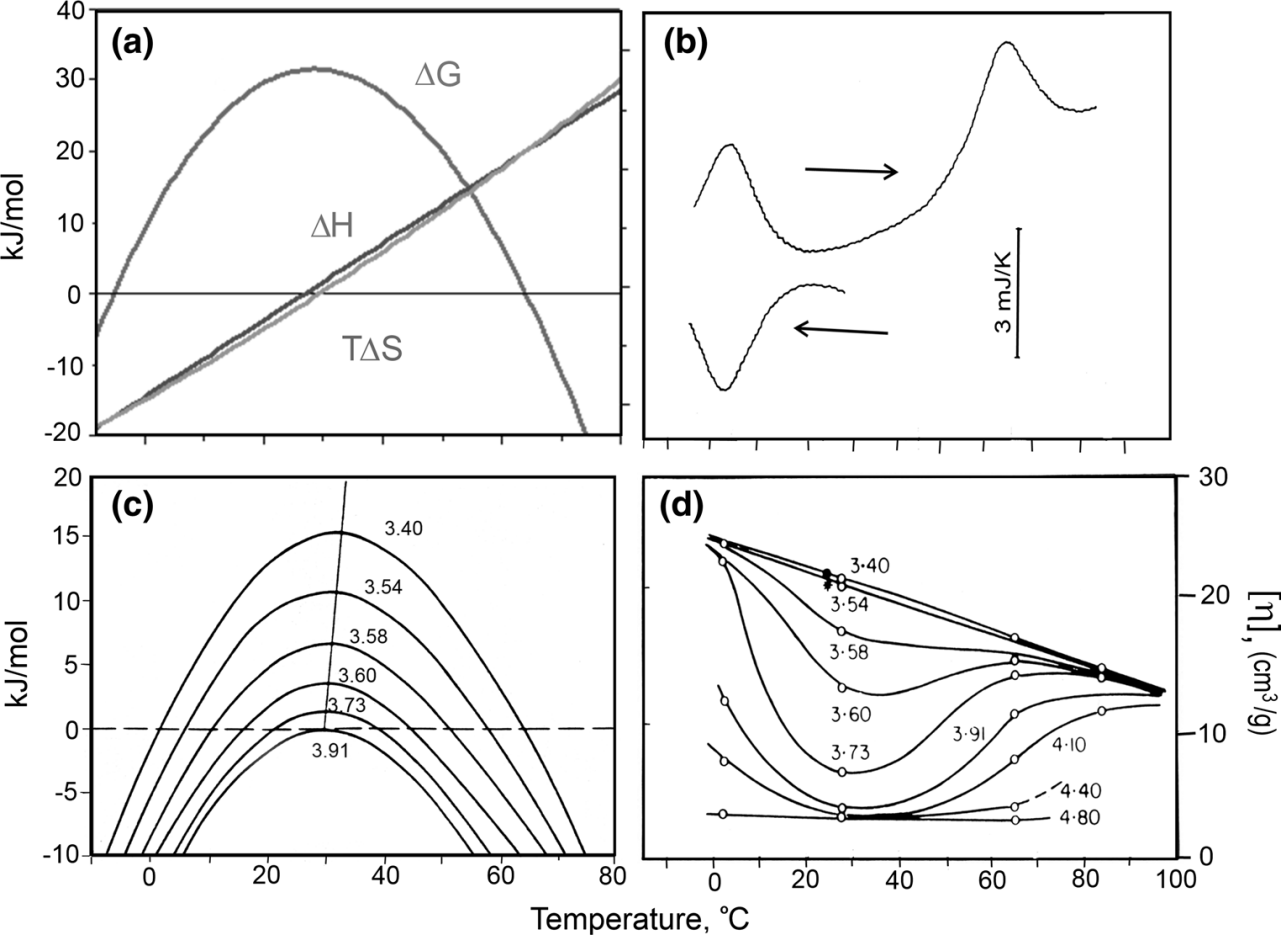
**a** The temperature dependencies of the enthalpy, entropy factor and Gibbs energy of myoglobin unfolding. **b** The DSC recorded heat effects upon cooling the myoglobin solution and its consecutive heating. **c** The calculated Gibbs energy functions of myoglobin in solutions with different pH. **d** Intrinsic viscosity of myoglobin in the solutions with different pH (Privalov et al. 1986)

Bearing in mind that the heat capacity increment is a temperature derivative of the enthalpy (∆*C*_p_ = d∆*H*/d*T*), the positive heat capacity increment means that the enthalpy of globular protein unfolding depends on temperature. We see that in Fig. 4, with increase of the transition temperature (as a result of a pH rise), the area of the excess heat absorption peak increases. Thus, using the calorimetrically measured enthalpy and the heat capacity increment of the temperature-induced protein transition, ∆*H*(*T*_t_) and ∆*C*_p_, one can determine the enthalpy of protein unfolding for any other temperature as:

1 $$\Delta H(T) = \Delta H(T_{\text{t}} ) - \Delta C_{\text{p}} \times (T_{\text{t}} - T)$$

Considering temperature-induced unfolding as a two-state transition, the entropy of this process can be determined by dividing the enthalpy by the absolute temperature of this transition, while at any other temperature it can be determined as: (for details, see Privalov 2012):

2 $$\Delta S(T) = \Delta H(T_{\text{t}} )/T - \Delta C_{\text{p}} \times (T_{\text{t}} - T)$$

Thus, these two functions, the enthalpy and entropy of protein unfolding, decrease as the temperature goes down, and one can expect that at some temperature they should change sign (Fig. 4a).

It is remarkable that, while the enthalpy of protein unfolding, ∆*H*(*T*), is a linear function of temperature, the entropy factor, *T*∆*S*(*T*), is not quite linear. Therefore, these two functions change with temperature in an almost parallel fashion, but at temperatures close to physiological the enthalpy function slightly prevails over the entropy factor; however, at higher and lower temperatures the entropy factor starts to prevail. Their difference

3 $$\Delta H(T) - T\Delta S(T) = \Delta G(T)$$

(given in Fig. 4a on the expanded scale) is the Gibbs energy, which represents the work required to transfer protein from the folded to the unfolded state. This Gibbs energy is, therefore, usually regarded as *a* *measure of protein structure stability*. It appears then that at physiological temperatures protein structure is stable: however, on temperature increase or decrease the difference between the enthalpy and entropy factor reduces and then changes sign. Thus, the native protein structure becomes unstable both above and below this optimal temperature, which is in the physiological range. Specifically, the stability of myoglobin at physiological temperatures amounts to about 30 kJ**/**mol. At this temperature the energy of thermal motion reaches the value:

$${\text{RT}} = 8.3\;{\text{J/K}}\;{\text{mol}} \times (37 + 273)\quad {\text{K}} = 2.5\;{\text{kJ/mol,}}$$

i.e., the protein stability at this physiological temperature is one order of magnitude higher than the energy of thermal motion. This is enough for the protein to withstand the disruptive action of thermal motion. It is notable, however, that the stability of protein structure is not too high. It appears that globular proteins just do not need an excessive stability, but some flexibility of structure is required, perhaps for proper functioning. Hydrogen exchange studies of such proteins indeed show that their structure fluctuates at physiological temperatures, but these are just independent micro-unfoldings of its structure (Hvidt and Nielson 1966). *The most surprising result of this thermodynamic analysis is that protein stability decreases not only upon heating, but also upon cooling from physiological temperatures, and thus one should expect proteins to denature not only on heating, but also on cooling*.

### Cold denaturation of proteins

The denaturation of proteins upon heating has never been considered as something surprising: it always seemed to be a natural phenomenon, even when nothing was known about protein structure, and proteins were only supposed to be rather complex molecular constructs built to fulfill various sophisticated functions in the cells. According to Le Chatellier’s principle, any process induced by increasing temperature should proceed with heat absorption and thus with disordering of the considered system. Therefore, disruption of the native protein structure upon heating, the heat denaturation of proteins, appeared as an obvious effect: it should unfold upon heating because of the increase of dissipative forces and should proceed with an enthalpy and entropy increase. However, a decrease of protein stability upon cooling is something unexpected, since dissipative forces of thermal motion decrease with cooling (for a review, see Privalov 1990).

In contrast to the well-known heat denaturation phenomenon, cold denaturation has not been observed in everyday life. This is because according to thermodynamic predictions (Fig. 4a), it should occur at temperatures below the freezing point of aqueous solutions, while in a frozen aqueous solution the protein certainly could not change its conformation. The only way to see if a protein indeed might unfold upon cooling is, therefore, to supercool its aqueous solution. Aqueous solutions can be supercooled to rather low temperatures if they do not contain dust particles, which serve as centers of crystallization.

It is notable that thermodynamics predicts that the enthalpy and entropy of protein unfolding, when extrapolated to lower temperatures, should have a sign opposite to that at high temperature (Fig. 4a). Therefore, following Le Chatellier, one would expect that, while protein unfolding upon heating proceeds with heat absorption (i.e., the enthalpy and entropy increase), upon cooling the protein should unfold with heat release, i.e., with an enthalpy and entropy decrease. This is just what was found: upon cooling the protein solution, a peak of heat release appears (Fig. 4b). Thus, in contrast to heat denaturation, cold denaturation proceeds with negative enthalpy and, moreover, with negative entropy. *It appears therefore that cold denaturation results in an increase in order*!

Changing the environmental conditions of a protein, e.g., the pH of the solution, usually results in changing the protein’s stability. Based on calorimetric data, the calculated Gibbs energy functions of myoglobin in solutions with different pH values represent a number of parabolic functions (Fig. 4c). As with the decrease of protein stability with temperature increase, one would also expect a decrease of its stability with cooling. This was confirmed directly by measuring the intrinsic viscosity of myoglobin in aqueous solutions differing in pH (Fig. 4d). Upon heating the intrinsic viscosity of myoglobin increases significantly at temperatures around 60 °C, showing that the compact globular structure unfolds. However, the intrinsic viscosity of the myoglobin solution also increases upon cooling down to 0 °C, even more than it does upon heating. The last is not surprising because at low temperatures the unfolded polypeptide chain is less flexible and occupies a larger hydrodynamic volume than at higher temperatures.

**Fig. 5:**
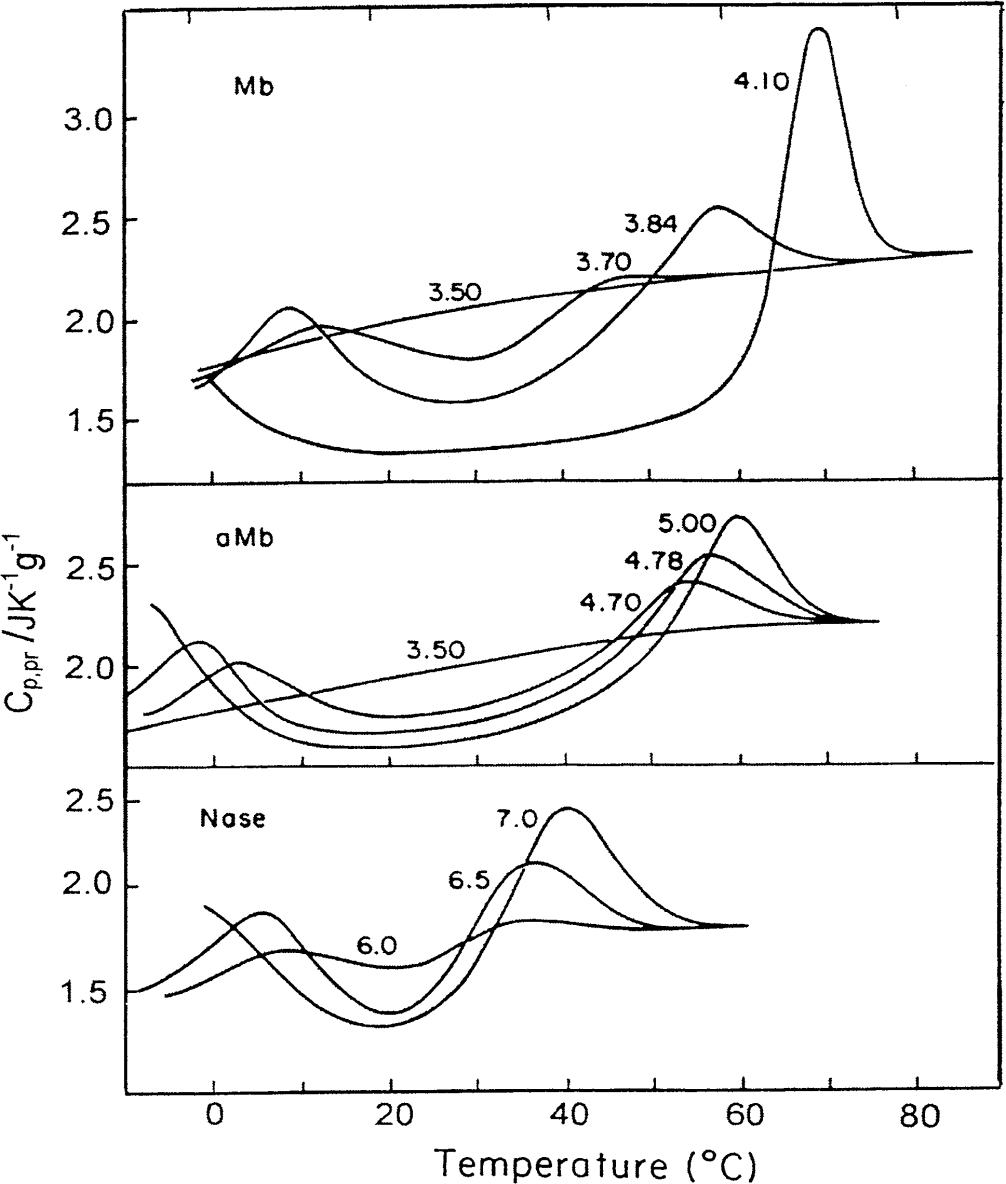
The DSC-measured partial heat capacity functions of myoglobin, apo-myoglobin and staphylococcal nuclease in solutions with different pH (Griko et al. 1988a, b; Privalov et al. 1986). With an increase of protein stability by raising the pH, the heat denaturation shifts to higher temperatures, while cold denaturation shifts to lower temperatures, as predicted by the thermodynamics (see Fig. 4c)

It is evident now that cold denaturation is a property specific for all globular proteins, although its observation is not easy since for most proteins it is expected to occur at too low temperatures (for a review, see Privalov 1990). *It can be observed experimentally only for proteins with a relatively large heat capacity increment, i.e., with a steeper enthalpy/entropy dependence on temperature.* Some of the calorimetrically studied examples are illustrated in Fig. 5.

The question to be answered is then: *why are the enthalpy and entropy of globular protein unfolding such steeply increasing functions of temperature, i.e., why does the unfolding of globular proteins proceed with such a considerable heat capacity increment?* The increase of conformational freedom of globular proteins upon unfolding is quite insufficient to explain the observed heat capacity increment typical for unfolding. However, unfolding of a globular protein results in exposure of its internal groups to water. These are mostly the apolar groups that are packed inside the native globular fold, thereby avoiding contact with water. The exposure of these groups to water, i.e., their hydration, is the key to understanding the folding/unfolding thermodynamics of globular proteins.

### Hydration effects

Dry proteins do not unfold either on heating or on cooling. Thus, the ability of proteins to denature upon heating and cooling seems to be caused by the presence of water. Hydration effects are usually studied by transfer of various compounds from the gaseous, liquid or solid phases into water. This led to understanding that the work required for transfer of various groups into water, which is the Gibbs energy of transfer, differs qualitatively for polar and nonpolar groups: it is negative for the highly soluble polar groups and is positive for the poorly soluble apolar aliphatic groups: correspondingly, the first are called *hydrophilic* and the second *hydrophobic*. Most surprising was the finding that *transfer of apolar groups from the condensed state into water at room temperature* (25 °C) *proceeds without noticeable heat effect,* *but with a significant heat capacity increment* (for review, see Privalov and Gill 1988). Thus, at *T*_H_ = 25 °C = 298.2 K the positive Gibbs energy of their transfer is due to the entropy, which should be large and negative:

4 $$\Delta S(T_{\text{H}} ) - (\Delta H - \Delta G)/T_{\text{H}} = \Delta G/T_{\text{H}} < 0$$

Negative entropy means an increase of order. It appears therefore that the low solubility of apolar groups in water results from their ability to order the water. However, nature does not like to be ordered: therefore, water expels the apolar groups. This expelling action of water on nonpolar compounds was considered by Kauzmann as a *hydrophobic force* (Kauzmann 1959).

The notion of the hydrophobic force gained popularity because it explained formation of the compact structure of globular proteins, i.e., a structure having minimal exposed surface. Moreover, it solved another serious problem of protein folding, namely, the thermodynamically unfavorable loss of conformational entropy of the polypeptide chain upon its folding into a compact, highly ordered conformation. It thus appeared that the negative entropy of polypeptide chain folding is compensated by the positive entropy of dehydration of the apolar groups of the chain on their removal from water. However, this elegant hydrophobic concept was later shaken by the discovery of cold denaturation.

**Fig. 6:**
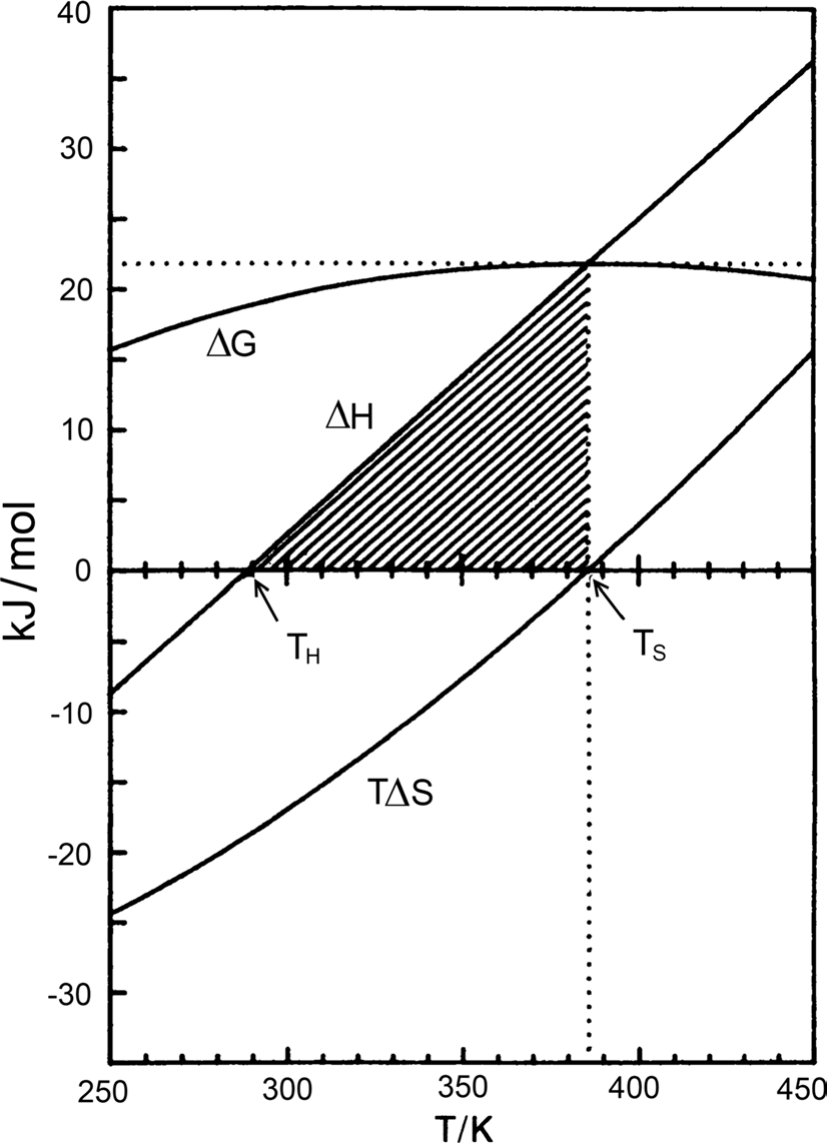
Thermodynamics of a liquid hydrocarbon dissolving into water, assuming a constant heat capacity change (Privalov and Gill 1988)

Indeed, an increase of the water order in the presence of apolar groups means an increase of hydrogen bonding between the water molecules, and this should result in a significant negative enthalpy effect. However, the enthalpy of transfer of nonpolar groups of protein into water is negligibly small at room temperature. This means that the enthalpy of water ordering is somehow balanced by the enthalpy of some other process. This other process can only be the disruption of van der Waals interactions between the apolar groups tightly packed in the protein interior. In Fig. 6 this temperature, at which the enthalpy of hydration of apolar groups is balanced by the enthalpy of their van der Waals interactions, is indicated as *T*_H_. At lower temperatures the increasing magnitude of the negative enthalpy of water ordering overrides the positive enthalpy of protein group associations. In contrast to the enthalpy, the entropy factor of apolar group transfer into water is negative at *T*_H_. With temperature increase it decreases in magnitude, and at temperature *T*_s_  ≈  112 °C it becomes zero.

It is notable that ∆*S* = 0 is a condition of the Gibbs energy extremum, since ∂*G*/∂*T* = −∆*S*. Thus, the maximal value of the Gibbs energy of transfer of an apolar compound into water is provided entirely by the enthalpy of their separation, i.e., by the enthalpy of van der Waals interactions between the apolar groups at this extremal temperature, *T*_S_. As the temperature decreases from *T*_S,_ the value of the Gibbs energy of transfer decreases both because the positive enthalpy of transfer decreases and because the negative entropy of transfer increases in magnitude.

Thus, the enthalpy and entropy of protein unfolding are strongly increasing functions of temperature, and this is because protein unfolding proceeds with a significant heat capacity increment. *This positive heat capacity increment is provided by the hydration of exposed nonpolar groups since the heat capacity effect of hydration of polar groups is negative* (for details, see review Makhatadze and Privalov 1995). Assuming that the heat capacity effect of hydration of apolar groups is temperature independent and taking the protein at temperature *T*_s_ as a standard state, the enthalpy and entropy of protein denaturation at temperature *T* can be represented to a first approximation as:

5 $$\Delta_{N}^{U} H(T) = \Delta_{N}^{U} H(T_{\text{S}} ) - \Delta_{N}^{U} C_{\text{p}} (T_{\text{S}} - T)$$

6 $$\begin{aligned} &\Delta_{N}^{U} S(T) = \Delta_{N}^{U} S(T_{\text{S}} ) - \Delta_{N}^{U} C_{\text{p}} \ln (T_{\text{S}} / T) \hfill \\ &\quad \approx \Delta_{N}^{U} S(T_{\text{s}} ) - \Delta_{N}^{U} C_{\text{p}} \left( {\frac{{T_{\text{S}} - T}}{T}} \right) {+} \Delta_{N}^{U} C_{\text{p}} \times \frac{1}{2} \times \frac{{(T_{\text{S}} - T)^{2} }}{T} \hfill \\ \end{aligned}$$

where $\Delta_{V}^{U} H(T_{\text{s}} )$ and $\Delta_{V}^{U} S(T_{\text{s}} )$ are the enthalpy and entropy of protein unfolding in the absence of hydration effects. Thus, to a first approximation, the Gibbs energy of stabilization of the native protein state becomes:

7 $$\begin{aligned} &\Delta_{N}^{U} G(T) = \Delta_{N}^{U} H(T) - T\Delta_{N}^{U} S(T) \hfill \\ &\quad \approx \Delta_{N}^{U} H(T_{\text{s}} ) - T\Delta_{N}^{U} S(T_{\text{S}} ) - \Delta_{N}^{U} C_{\text{p}} \times \frac{1}{2} \times \frac{{(T_{\text{S}} - T)^{2} }}{T} \hfill \\ \end{aligned}$$

**Fig. 7:**
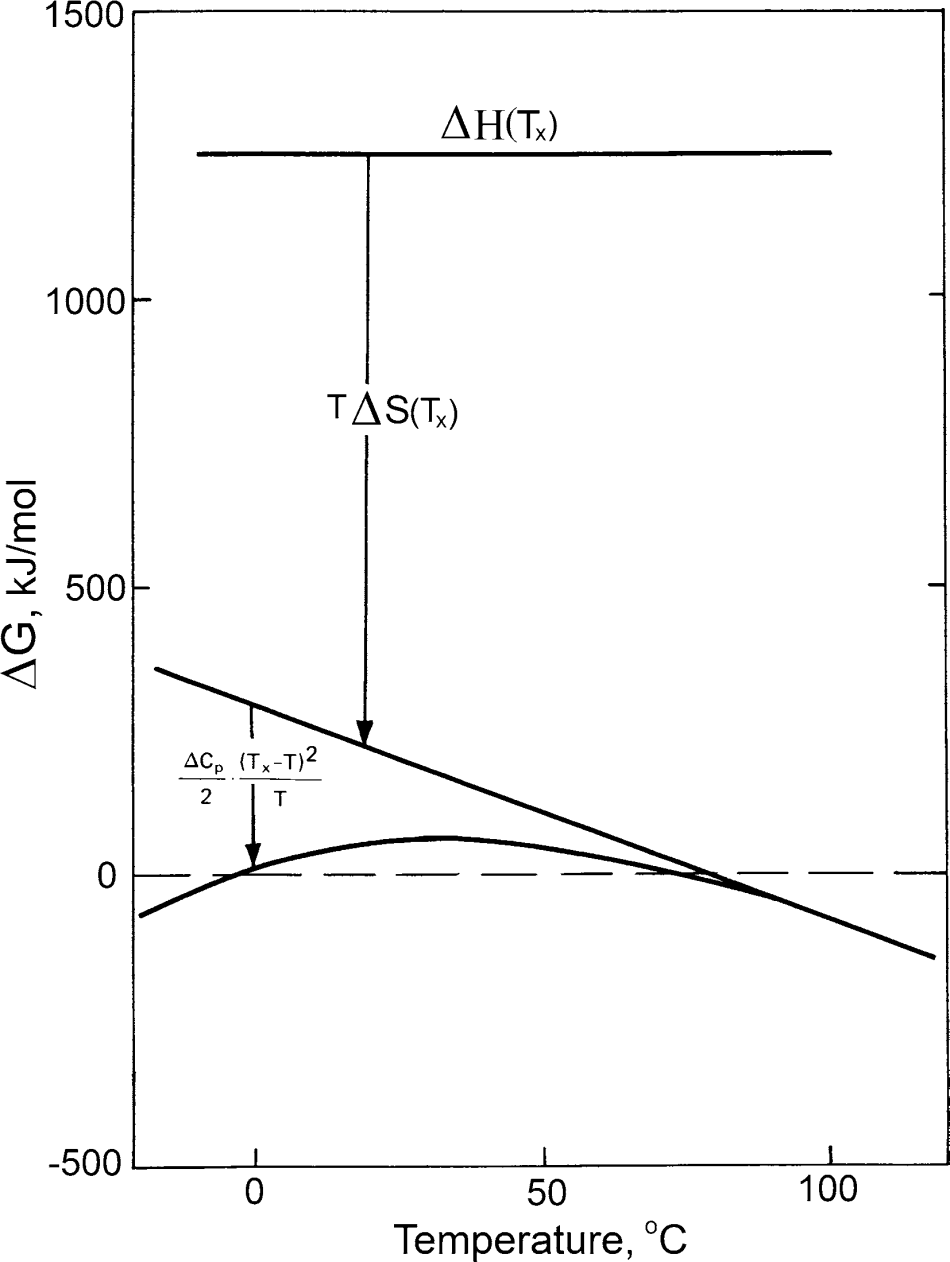
Contribution of the dissipative force [*T*∆*S*(*T*_x_)] and the water solvation effect [∆*C*_p_/2 (*T*_s_ − *T*)^2^/*T*] to the stabilization of an abstract globular protein (Privalov and Gill 1988)

Only the first term in Eq. (7), which represents the total enthalpy of hydrogen bonding and van der Waals interactions in a protein, is positive. The second term, which represents the action of dissipative forces, is negative and increases in magnitude with temperature rise. The third term represents the effect of hydrating the nonpolar groups: this term is zero at *T*_s_ but rapidly increases in magnitude as the temperature is lowered (Fig. 7). It appears, therefore, that this third term is responsible for the cold denaturation of proteins. The main importance of this term is, however, that it *optimizes the stability of globular proteins at physiological temperatures, making protein structure at this temperature flexible enough for its proper functioning*.

## The collagen–water partnership

### Forces stabilizing the collagen triple helix

Collagen is an extreme example of a fibrillar protein in having a very large exposed surface area per unit volume. It is also the most abundant protein in the animal kingdom, being the main component of skin, tendon, cartilage and bone.

The primary structure of all collagens is very simple: a repeat of a standard triplet, Gly-X-Y-, so that every third residue is glycine, while X and Y are—with high probability—proline or hydroxyproline:

$${\text{Gly-X-Y-Gly-X-Y-Gly-X-Y-Gly-X-Y-Gly-X-Y-}}{\text{Gly-X-Y-}}$$

Hydroxyproline (Hyp) appears as a result of post-translational modification of proline if it is in the Y position of the triplet.

**Fig. 8:**
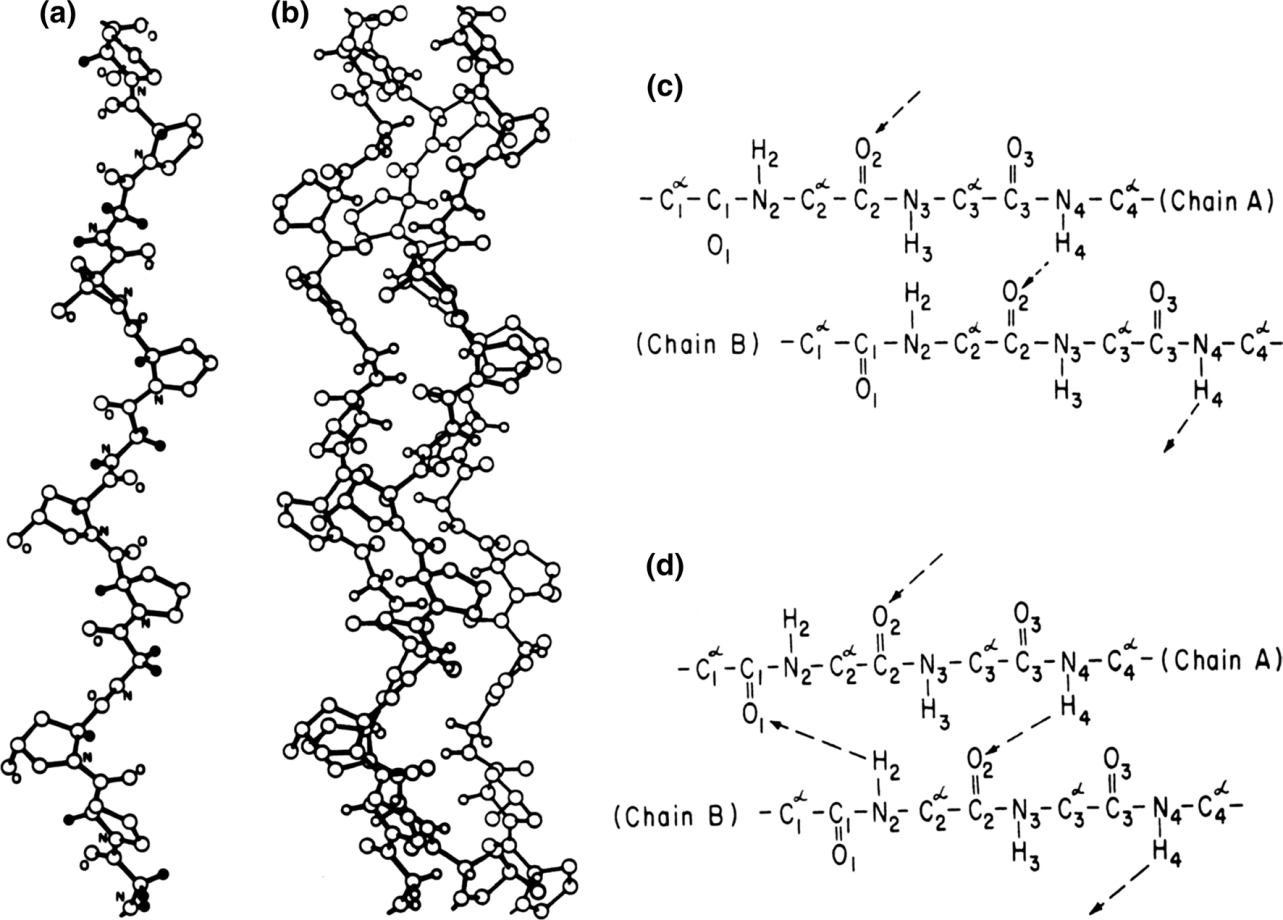
**a** A single strand of the repeated -Gly-Pro-Hyp- sequence in the poly-l proline conformation. **b** The three-stranded coiled coil. **c** The one-bonded model (Rich and Crick 1955); **d** the two-bonded mode (Ramachandran and Kartha 1955)

The polypeptide chain of collagen is highly flexible, and in aqueous solution it forms a random coil. However, three similar polypeptide chains of collagen can associate forming a poly-l-prolyl super-helix (Fig. 8a, b), a very stable rope-like construction suitable for transfer of mechanical stress over long distances in the tissues. The question is then: what are the forces stabilizing this collagen superhelix?

Theoretical consideration of possible conformations of the poly-l-prolyl polypeptide led to two alternative coiled-coil models of collagen, with somewhat different packing and different amounts of internal hydrogen bonds stabilizing the helical structure: the less tight one-bonded model suggested by Rich and Crick (1955) and the tighter two-bonded model suggested by Ramachandran and Kartha (1955) (Fig. 8c, d). According to the one-bonded model, the collagen polypeptides in the poly-l proline conformation are connected by one hydrogen bond per triplet, between the amide group of the glycine, in the first position, and the carbonyl oxygen of a residue in the second position (X) of the triplet. According to the two-bonded model, there is an extra hydrogen bond between the carbonyl oxygen of the glycine and the amide NH of a residue in the second position of the triplet, provided this position is not occupied by an imino acid residue (Pro or Hyp). Clearly, the number of these second H-bonds is less than one per triplet and decreases with a rise of the imino acid content in collagen—assuming that imino acid residues occupy the second (X) and third (Y) position in the triplet with fairly equal probability.

### Stability of the collagen triple helix

**Fig. 9:**
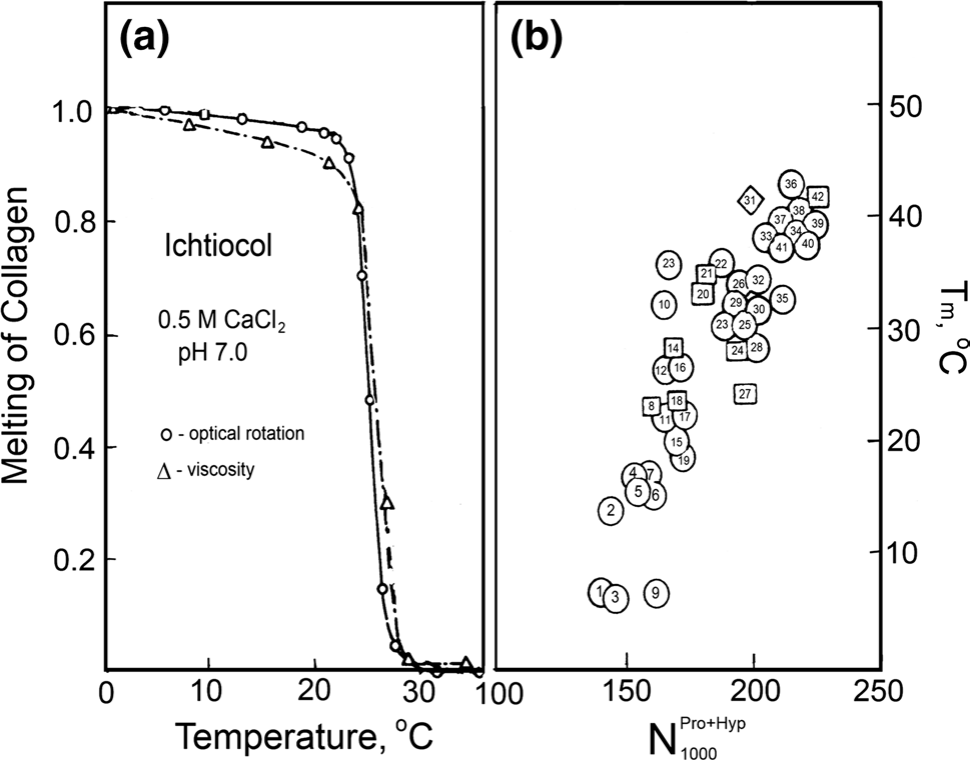
**a** Breakdown of the collagen structure upon heating observed by drastic changes in the intrinsic viscosity and optical rotation (von Hippel 1967). **b** Plot of collagen melting temperature in salt-free solution at pH 3.7 versus the total imino acid content per 1000 residues (for details, see Privalov 1982)

A striking feature of collagens is that, on increase of temperature above some critical level, their regular rigid structure breaks down over a rather short temperature range, the chains separating into three independent random coils (Fig. 9a).

It is remarkable that collagens obtained from different sources do not differ essentially in their conformation but differ significantly in their thermal stabilities: collagen stability increases with the imino acid content, i.e., proline plus hydroxyproline (Fig. 9b). The increase of collagen thermostability with imino acid content was explained by the reduced number of conformations caused by the presence of the pyrrolidine ring. According to Harrington (1964), if the entropy gain per residue on disruption of the collagen structure is ∆*S*_res_ ≈ 17 J/K mol, for an imino acid restricted by the pyrrolidine ring it is zero. Thus, the entropy of melting of a collagen block consisting of 1000 residues should be:

8 $$\Delta_{\text{m}} S_{1000} = \Delta S_{\text{res}} \times (1000 - N_{\text{pro}} ),$$

where *N*_pro_ is the number of hydroxyprolyl plus prolyl residues per 1000 total residues in the collagen. As for the enthalpy of collagen melting, it should be different for the two existing models. For the one-bonded model it should be:

9 $$\Delta_{\text{m}} H^{\text{I}}_{1000} = 333 \times H^{\text{H}} \,,$$

where ∆*H* ^H^ is the enthalpy of disruption of one peptide hydrogen bond.

For the two-bonded model it should be:

10 $$\Delta_{\text{m}} H^{\text{II}}_{1000} = (666 - N_{\text{pro}} ) \times \Delta H^{\text{H}} ,$$

where *N*_pro_ is the number of pyrrolidines occurring in the second position in the triplet, per 1000 total residues. Assuming that at the midpoint of collagen melting ∆_m_ *G* = ∆_m_ *H* − *T*_m_∆_m_ *S* = 0, and thus *T*_m_ = ∆_m_ *H*/∆_m_ *S*, one finds for the one-bonded model:

11 $$T_{\text{m}} = 333 \times \Delta H^{\text{H}} /[(1000 - N_{\text{pro}} ) \times \Delta S_{\text{res}} ],$$

and for the two-bonded model:

12 $$T_{\text{m}} = (666 - N_{\text{pro}} ) \times \Delta H^{\text{H}} /[(1000 - N_{\text{pro}} ) \times \Delta S_{\text{res}} ]$$

Thus, according to both models the melting temperature of collagen should increase with an increase of the imino acid content. As for the enthalpy, for the one-bonded model it should not change with an increase of the imino acid content; thus, it should not change with an increase of the collagen thermostability. For the two-bonded model the enthalpy should decrease with an increase of the imino acid content, i.e., with an increase of the thermostability of collagens. Thus, *the* *calorimetric study of collagen melting became crucial for checking the existing concepts of collagen structure stabilization.*

### Calorimetry of collagen melting

**Fig. 10:**
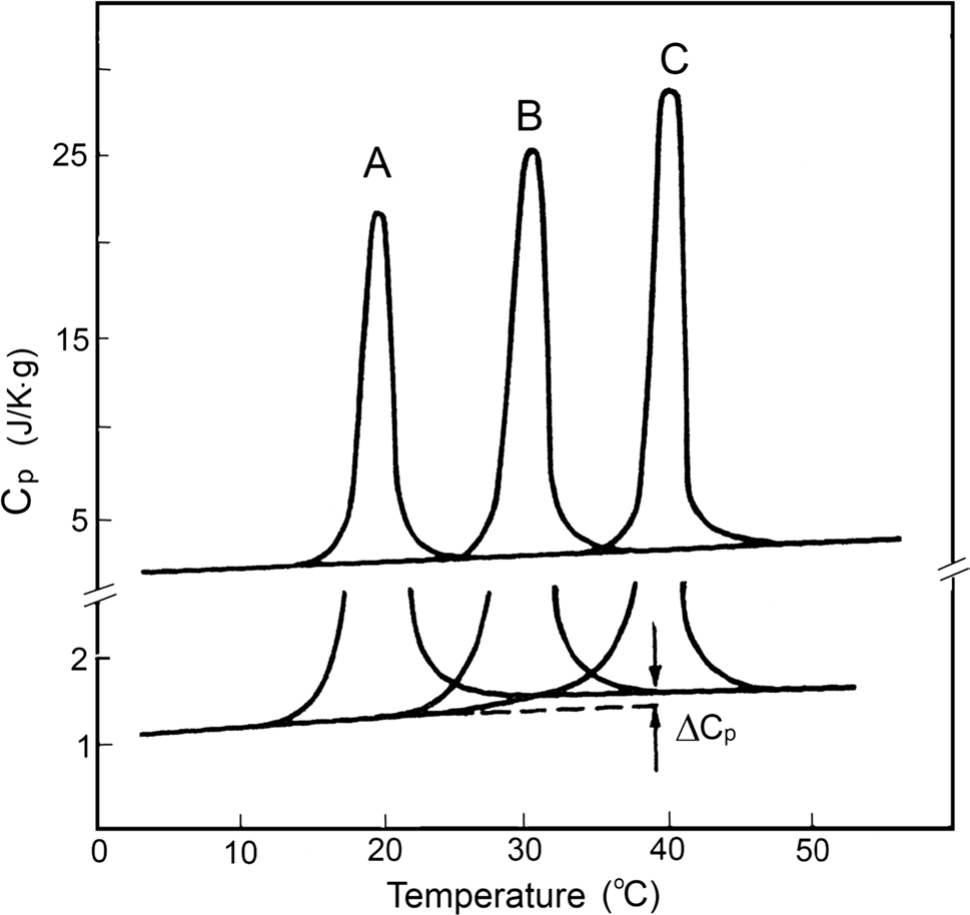
Temperature dependence of the partial specific heat capacity of cod (*A*), pike (*B*) and rat (*C*) skin collagens in pH 3.5 salt-free solution. A fragment at a magnified scale is presented under melting profiles to demonstrate the denaturational heat capacity increment, (Privalov 1982)

The very first calorimetric studies of melting collagens from various sources showed that temperature-induced breakdown of their helical structure proceeds with extensive heat absorption, which increases with the rise of their thermostability (Privalov and Tiktopulo 1970). Most surprising was that *‘melting’ of collagen proceeds with a very small, if any, heat capacity increment* (Fig. 10; Table 2). Therefore the observed increase of the heat effect of collagen melting, i.e., the enthalpy of collagen structure breakdown, cannot be explained by the simple dependence of the transition enthalpy on temperature expressed by Kirhoff’s relation: ∆*C*_p_ = d∆*H*/d*T*. It appeared, therefore, *that neither of the suggested models explains the energetic basis of the collagen superhelix.*

**Table 2:** Structural characteristics of collagens and thermodynamic parameters of their unfolding/dissociation (Privalov 1982)

No.	Source	(*N*_1000_)		*T*_t_ °C	∆*H*_t_ (J/mol_res_)	∆*S*_t_ (J/K mol_res_)
		Pro	4-Hyp*			
1	Cod skin	103	58	15	4100	14.0
2	Halibut	113	66	18	4490	15.4
3	Frog skin	106	68	31.2	4880	15.6
4	Pike skin	134	73	30.0	5240	17.3
5	Carp swim bladder	121	84	34.3	5150	16.7
6	Rat skin	112	115	39.7	6450	21.5
7	Sheep skin	133	97	39.0	6310	20.2

3-Hydroxyproline is regarded as Pro. (*N*_1000_)—imino acid content per 1000 residues

Furthermore, the absence of a heat capacity increment upon collagen unfolding also showed that, in contrast to globular proteins, *there are no apolar groups in the collagen triple helix that might be exposed to water upon its unfolding*.

**Fig. 11:**
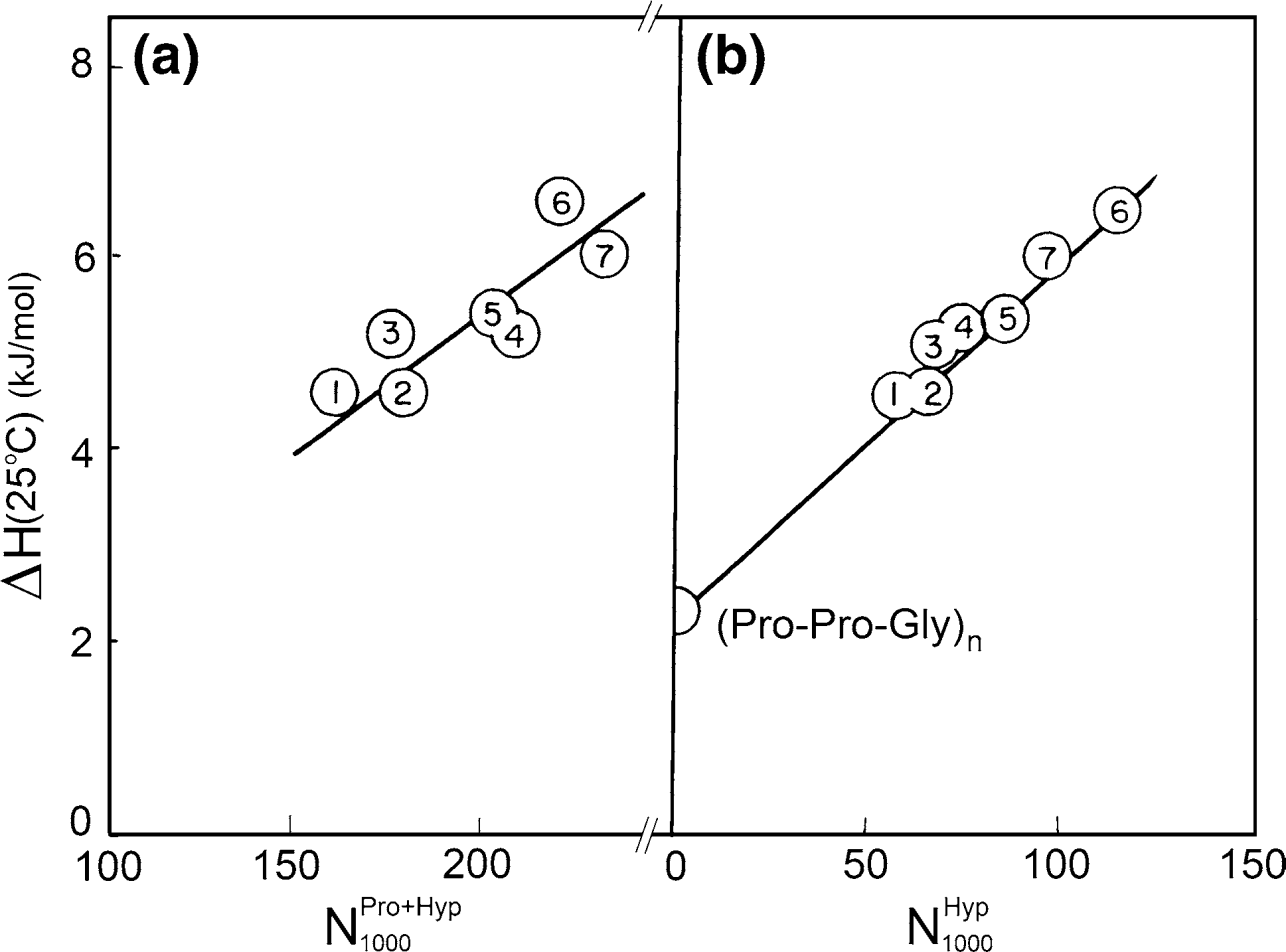
Plot of the melting enthalpy of collagens (per mole of residue values, extrapolated to 25 °C) versus **a** the total prolyl and hydroxyprolyl content and **b** only hydroxyprolyl content in the helical parts of various species: *1* cod skin, *2* halibut, *3* frog skin, *4* pike skin, *5* carp swim bladder, *6* rat skin and *7* sheep skin (Privalov 1982)

Analysis of the melting enthalpy of a number of collagens differing in their thermostabilities showed that it correlates better with the content of hydroxyproline than proline (Fig. 11). This, however, is true only for the collagens of vertebrates in which the hydroxyproline is only in the third position in the triplet (Burjanadze 1979).

The fact that the melting enthalpy of collagens increases with the rise in their thermostability and this closely correlates with the hydroxyproline content indicates clearly *that the main stabilizing effect comes not from the rigidity of the pyrrolidine ring but from hydroxyproline specifically, if this hydroxyproline is in the third (Y) position in the triplet.* It was unclear, however, how this hydroxyl group could induce a significant increase in the melting enthalpy and entropy of the collagen coiled coil because the hydroxyl groups of prolines in the third position in the triplet are exposed to water, i.e., they are unable to form hydrogen bonds within the triple helix (Burjanadze 1979). But these exposed hydroxyl groups of prolines *are* able to interact with the water surrounding the collagen superhelix. Bearing in mind the well-known tendency of water molecules to cooperate with their neighbors, one would expect that *the hydroxyproline residues in these positions along the collagen chain initiate an extensive cooperative network of water hydrogen bonding that envelops the collagen* (Privalov 1982). *This water might be responsible for the exceptionally large enthalpy of collagen “melting,” many times exceeding the enthalpy of globular protein* *denaturation.* If so, one would expect to see this bound water crystallographically.

### Crystallography of collagen

**Fig. 12:**
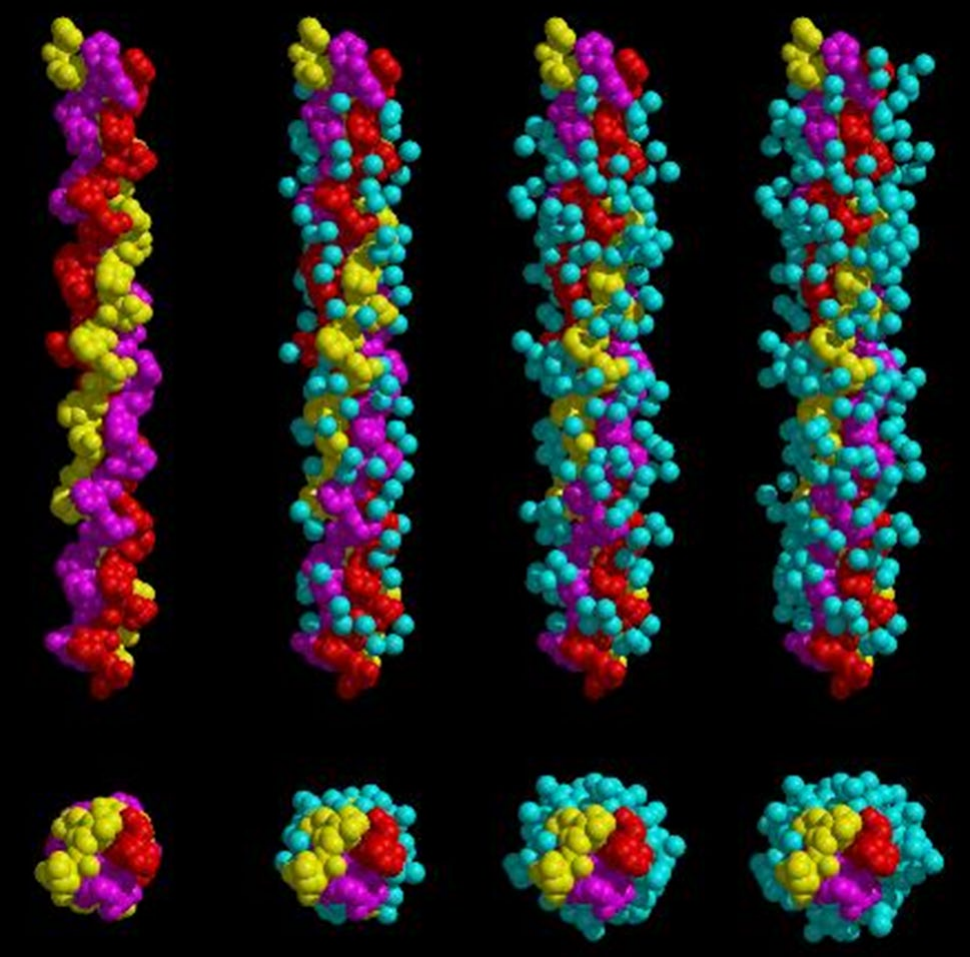
Crystal structure of a synthetic (Pro-Hyp-Gly) collagen-like triple helix. The three stands are in yellow, red and magenta and layers of fixed water molecules (in cyan) cover the triple-helix. Repetitive patterns of water bridges link oxygen atoms both within a single peptide chain, between different chains and even between different triple helices. Overall, the water molecules are organized in a semi-clathrate-like structure that surrounds and interconnects triple-helices in the crystal lattice (Bella et al. 1995)

The earlier attempts at collagen crystallization were not amenable to a detailed investigation of its structure. The path to the molecular details of the collagen triple helix has been through collagen model peptides, which have yielded high-resolution X-ray structures. They provided the first visualization of the elaborate water network that surrounds collagen molecules (Bella and Berman 1996; Bella et al. 1995). Water molecules are seen to bridge carbonyl C=O and Hyp–OH groups, and a repetitive network of these water patterns is strung along the chain (Fig. 12). An increasing number of high-resolution structures have confirmed that extended water networks are an inherent feature of all collagen triple-helix peptide crystal structures (Berisio et al. 2001).

## Stability and flexibility of collagen structure

**Fig. 13:**
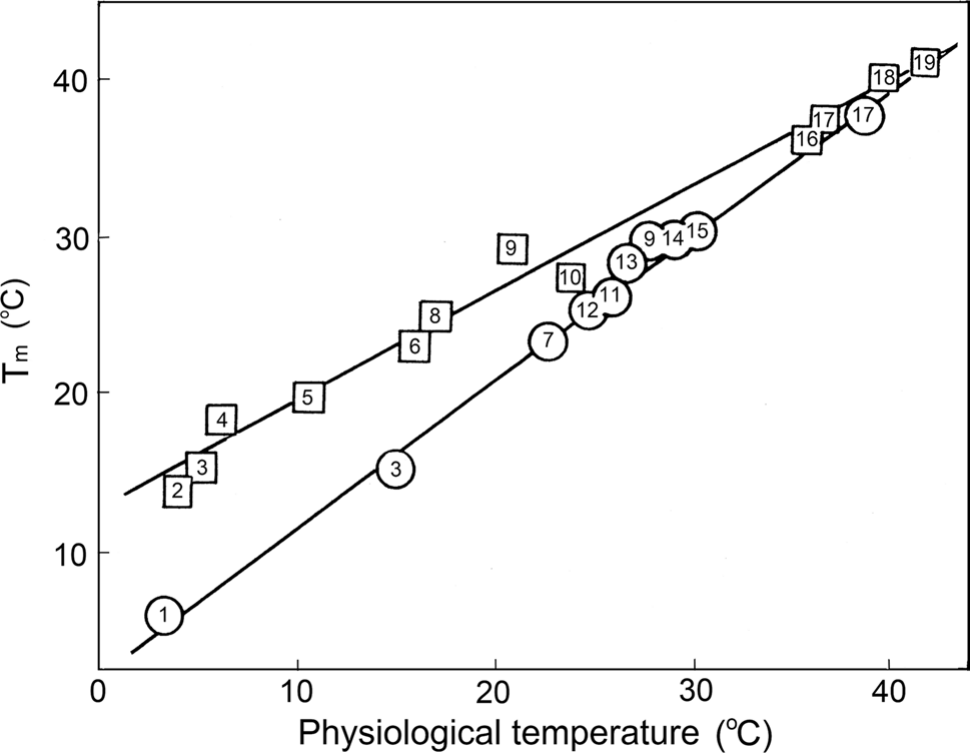
Plot of the melting temperature of collagens from various species versus the average physiological temperature of the species (*squares*). Also plotted are the upper limits for the physiological temperatures of the different species (*circles*). *1* Ice fish; *2* *Antimora* (violet cod); *3* cod; *4* *Alepocephalus* (slickhead fish); *5* whiting; *6* *Allolobaphora caliginosa* (earthworm); *7* earthworm; *8* flatfish; *9* *Cyprinus carpio* (carp); *10* butterfly fish; *11* tuna; *12* *Rana tempararia* (frog); *13* *Aurelia coerula* (jellyfish); *14* *Rana ridibunda* (frog); *15* *Helix aspersa* (snail); *16* rat; *17* human; *18* pig; *19* chicken (Privalov 1982)

The question of paramount importance is now: why do collagens from different species differ in stability? Certainly, the thermostability of a collagen should be high enough to preserve its structure from the destructive action of thermal motion at physiological temperatures. Expressed by the melting temperature, the thermostability of mammalian collagen indeed appears a few degrees above their physiological temperature, i.e., above 37 °C. This collagen will, therefore, also be stable for the polar fishes with their much lower physiological temperature. Surprisingly, however, the collagen of polar fishes has a much lower melting temperature (Fig. 13). It appears then that the melting temperature of collagens closely correlates with the physiological temperature of the species from which it has been isolated.

The correlation between physiological temperature and collagen thermostability, expressed in their melting temperatures, can be explained by assuming that *some definite level of flexibility of protein structure is required for its functioning in living species.*

The flexibility of protein structure is usually judged by the rate of hydrogen exchange and is expressed in the Gibbs energies of its micro-unfoldings, ∆*G* ^mic^, (for details, see Privalov 2012), while the physical measure of the stability of protein structure is the work required for its macroscopic unfolding, i.e., the macroscopic Gibbs energy of unfolding, ∆*G* ^mac^. These physical characteristics of the stability and flexibility of collagens from various species differing in their physiological temperatures are presented in Table 3. It appears that for species having very different physiological temperatures, the Gibbs energies of microscopic unfolding at their physiological temperatures are remarkably similar, unlike those of macroscopic unfolding (Privalov et al. 1979). *Thus, the flexibility of the collagen structure is a property important for its functioning, and this is achieved by the very specific interaction of collagen with the water forming the frame around the collagen superhelix*.

**Table 3:** Gibbs energies of macro- and micro-unfolding of collagens at standard temperature (25 °C) and at physiological (ph) temperature (Privalov et al. 1979)

Organism	*T*_ph_ (°C)	∆*G* ^mac^ (J/mol)		∆*G* ^mic^ (kJ/mol)	
		At 25 °C	At *T*_ph_	At 25 °C	At *T*_ph_
Cod	5.0	−80	204	6.0	8.0
Pike	14.0	88	292	10.5	11.0
Frog	19.0	120	219	9.0	9.5
Carp	18.0	180	308	11.0	11.0
Rat	35.0	323	405	11.7	11.1

## The DNA double helix

### Forces stabilizing the DNA double helix

According to the Watson-Crick model, an important role in DNA double helix stabilization was played by the hydrogen bonds between complementary bases: two between adenine and thymine and three between cytosine and guanine. One would expect, therefore, that an increase of the CG base pair content should lead to an increase in DNA stability. This is just what was found experimentally: the thermal stability of the DNA duplex indeed increases with a rise in CG content. This experimental fact was considered as a strong argument for the correctness of the Watson-Crick DNA model. It became evident that the greater stabilizing effect of the GC base pair results from its extra hydrogen bond and therefore from a larger enthalpic contribution of this base pair. This explanation for the observed increase of DNA stability with increase of GC content became conventional in all textbooks of Biochemistry and Molecular Biology. However, to justify this conclusion it was highly desirable to measure the enthalpy of base pairing calorimetrically.

The first calorimetric attempts to measure the enthalpy of DNA melting were rather confusing: various authors gave very different numbers in the range between 35 and 60 kJ/mol-bp, but all authors agreed that the enthalpy of CG base pairing significantly exceeds that of AT base pairing (Breslauer et al. 1986; Gotoh and Tagashira 1981; SantaLucia and Hicks 2004; Sugimoto et al. 1996). However, as shown below, the appearance of nano-calorimetry led to a complete reconsideration of the energetic basis of the DNA double helix.

### Calorimetric studies of DNA

**Fig. 14:**
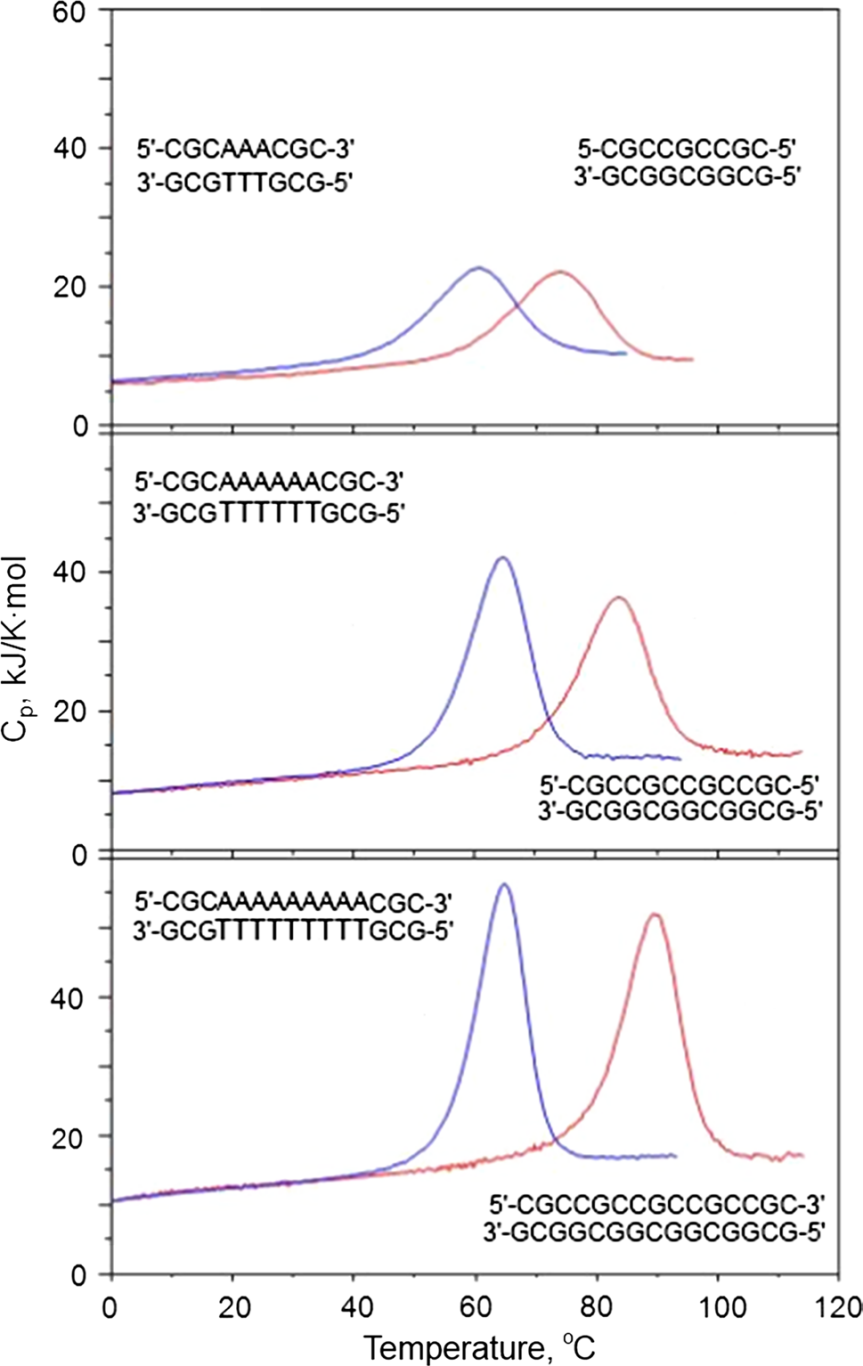
Comparison of the partial molar heat capacities of 9, 12 and 15 base pair CG duplexes (*red*) and the same length duplexes including AT pairs (*blue*), all at the identical molar concentration of 283 µM in 150 mM NaCl, 5 mM Na-phosphate, pH 7.0 (Vaitiekunas et al. 2015)

**Fig. 15:**
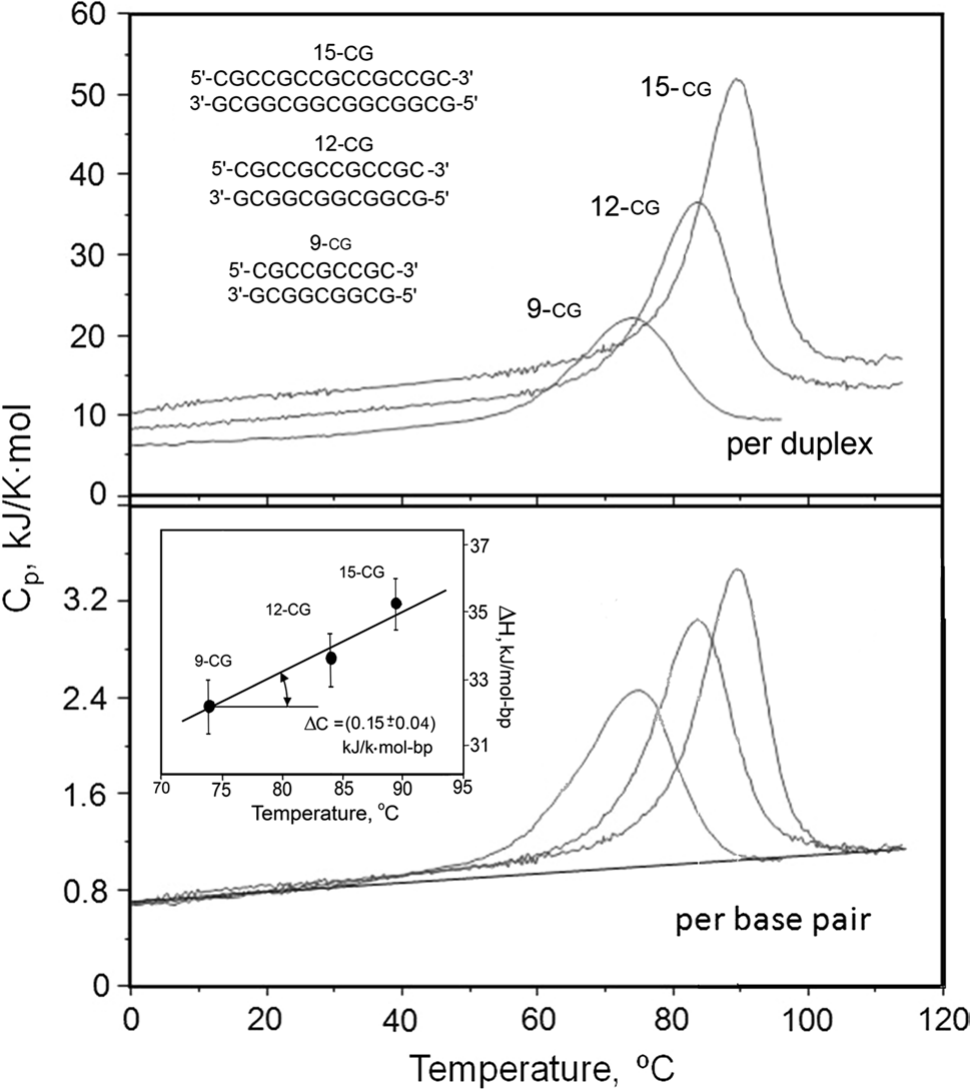
The partial heat capacity functions of the three considered CG DNA duplexes calculated per mole of duplex (molar heat capacity, *upper panel*) and per mole of base pair (specific molar heat capacity, *lower panel*), all measured at the same molarity, 230 μM, of the duplexes in 150 mM NaCl, 5 mM Na-phosphate, pH 7.4. *Inset* the dependence of the excess enthalpy on the transition temperature, the slope of which gives an estimate of ∆*C*_p_ (Vaitiekunas et al. 2015)

Nano-calorimetric studies of the temperature-induced melting of DNA duplexes showed that, as expected, duplexes consisting only of CG base pairs melt at higher temperatures than duplexes of the same length also containing AT base pairs (Fig. 14). Unexpected, however, was the finding that duplexes containing AT base pairs melt with a larger heat effect. Furthermore, it also appeared that the heat of melting the DNA duplex increases with the melting temperature, thus suggesting that dissociation of the DNA strands is accompanied by a definite heat capacity increment (Fig. 15). Calculated per base pair, this heat capacity increment appeared to be about 0.15 kJ/K mol-bp and identical for the CG and AT base pairs. Calculated per gram, this heat capacity increment amounts only to 0.18 J/K g. It is therefore significantly lower than the heat capacity increment specific for globular proteins, which is between 0.5 and 0.7 J/K g (Makhatadze and Privalov 1995). The temperature dependence of the enthalpy and entropy of DNA unfolding is therefore much more modest than that of proteins, and they do not change sign on lowering the temperature; correspondingly, *one cannot expect that the DNA double helix will unfold upon cooling, as occurs for globular proteins*.

Calorimetric studies of DNA duplexes of various lengths consisting only of CG base pairs showed that the contributions of individual base pairs to the enthalpy of duplex unfolding appear to be additive (Vaitiekunas et al. 2015). Therefore, dividing the total enthalpy of duplex dissociation by the number of constituent CG pairs in the duplex gives the enthalpic contribution of a single base pair. To extract the contribution of AT pairs from duplexes of mixed composition, one must first exclude the expected contribution of the two terminal CGC/GCG triplets from the measured total enthalpy of dissociation and then divide the remaining enthalpy by the number of AT pairs in the duplex:

13 $$\Delta H_{\text{AT}} (T) = \frac{{\Delta H_{\text{duplex}} (T) - N_{\text{CG}} \times \Delta H_{\text{CG}} (T)}}{{N_{\text{AT}} }}$$

The entropy of cooperative dissociation of a duplex can be determined at the melting temperature by dividing the DSC-measured heat of this cooperative processes by the absolute temperature and correcting for the concentration:

14 $$\Delta S^{\text{coop}} (T_{\text{t}} ) = \frac{{\Delta H_{\text{m}}^{\text{coop}} }}{{T_{\text{t}} }} + R\ln \left( {\frac{[N]}{2}} \right)$$

To determine the contribution of a single CG base pair to the conformational entropy of the helix, one must exclude the translational entropy (assumed to be ∆*S* ^trans^ = 34 J/K mol, see “Formation of macromolecular complexes”) from the total conformational entropy of a duplex consisting only of CG base pairs and divide the remaining entropy by the number of base pairs in the duplex, assuming their contributions are additive:

15 $$\Delta S_{\text{CG}}^{\text{conf}} (T) = \frac{{\Delta S_{\text{CG}}^{\text{tot}} (T) - \Delta S^{\text{trans}} }}{{N_{\text{CG}} }}$$

The entropic contribution of AT base pairs can be determined from the total entropy of dissociation of AT-containing duplexes by first excluding the translational entropy and also the contribution of the CG base pairs in that duplex before dividing by the number of AT pairs:

16 $$\Delta S_{\text{AT}}^{\text{conf}} (T) = \frac{{\Delta S_{\text{duplex}}^{\text{tot}} (T) - \Delta S^{\text{trans}} - N_{\text{CG}} \times \Delta S_{\text{CG}}^{\text{conf}} (T)}}{{N_{\text{AT}} }}$$

**Fig. 16:**
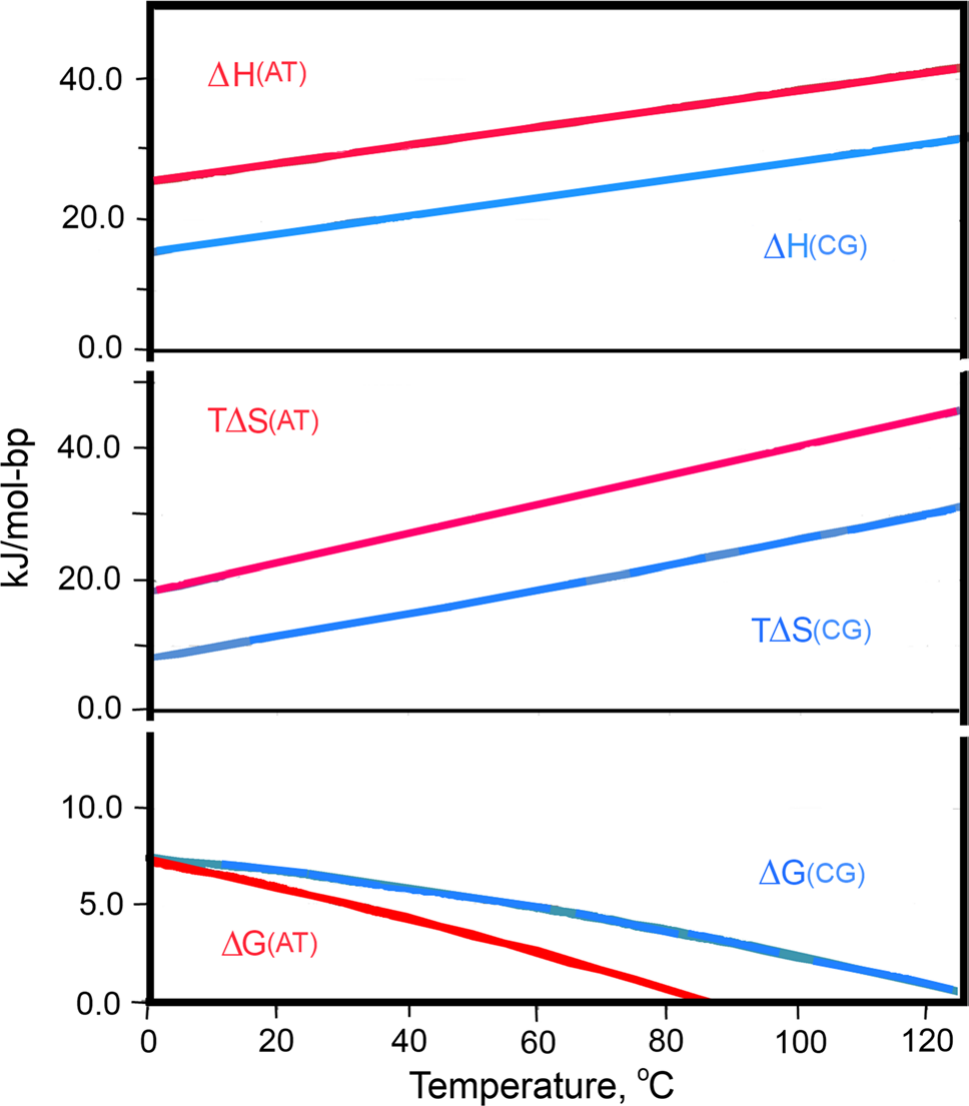
Contributions of CG and AT base pairs to the enthalpy (∆*H*), entropy factor (*T*∆*S*) and Gibbs energy (∆*G*) of the cooperative phase of DNA duplex dissociation. For more details, see Vaitiekunas et al. (2015)

The results of such an analysis of all studied DNA duplexes are summarized in Fig. 16. It shows that *the enthalpic contribution of the AT base pair to duplex stabilization significantly exceeds that of a CG pair at all temperatures* (for details, see Vaitiekunas et al. 2015). This was an absolutely unexpected conclusion. However, even more surprising was the finding that *the entropy contribution of the AT base pair is significantly larger than that of the CG pair.* It follows that the CG-rich DNA duplex is more stable than the AT-rich duplex not because the enthalpy of CG dissociation is larger than that of ATs, but because the entropy of its dissociation is lower. Alternatively, this could be stated as: *the AT*-*rich duplex is less stable than the CG*-*rich duplex because the entropy of AT dissociation is larger than the entropy of CG dissociation*.

### The water component of the DNA duplex

The larger enthalpic contribution to DNA dissociation of the AT pair relative to the CG pair certainly cannot be caused by differences in hydrogen bonding between the complementary bases since the AT pair has fewer such bonds than CG, nor can it be caused by differences in stacking interactions of the bases packed in the double helix, since these are quite similar for the two base pairs. Even more difficult to understand is why the entropy contribution of the AT base pair substantially exceeds that of the CG pair. These differences can be caused only by a component external to the DNA, i.e., by the water specifically bound by the AT base pair.

**Fig. 17:**
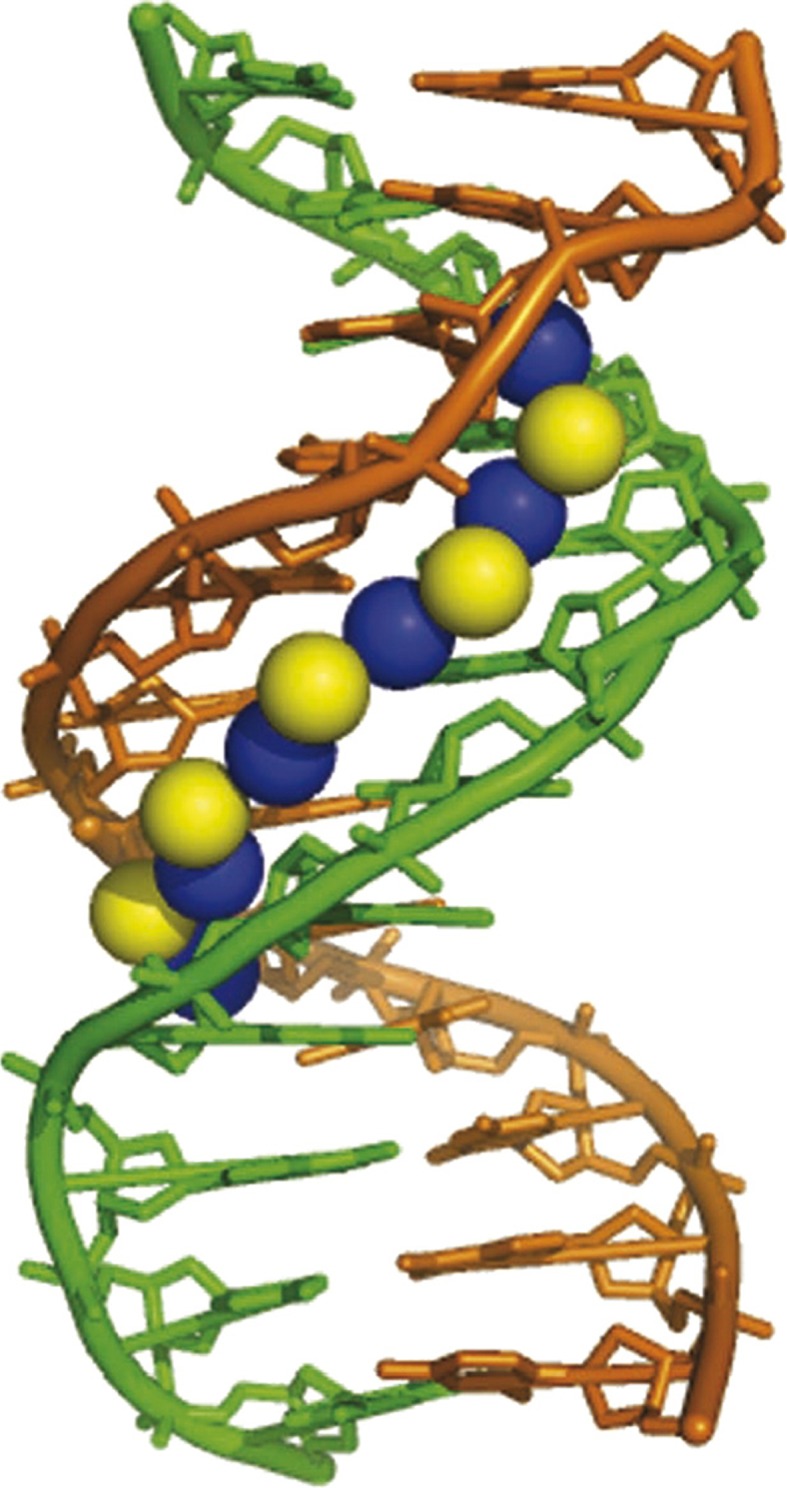
Display of primary (*blue*) and secondary (*yellow*) layers of the spine of water in the minor groove of the crosslinked dodecamer CGCGAATTCGCG, generated from the coordinates of NDB accession number BD0008 [reproduced from (Privalov et al. 2007)]

The existence of such bound water molecules has been observed crystallographically and by NMR as a spine in the minor groove of AT-rich DNA (Chiu et al. 1999; Drew and Dickerson 1981; Kopka et al. 1983). Furthermore, it was found that a secondary shell of water molecules runs along the groove in AT stretches, donating hydrogen bonds to the primary shell of oxygen atoms that assume the tetrahedral coordination characteristic of ice (Fig. 17).

It should be noted that water ordering in the minor groove of AT-rich DNA is provided not by apolar groups, as occurs in the case of proteins: in contrast, it is fixed by the polar groups of the AT pair, namely by N3 of A and O2 of T (Kopka et al. 1983; Shui et al. 1998), and is released upon dissociation of this pair. Judging by the excess entropy contribution of AT base pairing over CG pairing, which exceeds by almost two-fold that of melting ice (22 J/K mol), the AT-fixed water molecule affects the state of a number of surrounding water molecules. Thus, *one would expect that water ordering in the minor groove of DNA should depend on the mutual arrangement of AT base pairs and also on their orientations*. It thus appears that *the disposition of AT base pairs along the DNA and their mutual orientation should orchestrate water ordering in the minor groove* (Vaitiekunas et al. 2015).

An important feature of the enthalpy of DNA duplex unfolding/dissociation is that for all the considered DNA duplexes it increases linearly with temperature (Fig. 15). This means that *duplex unfolding proceeds with a definite heat capacity increment.* The origin of this heat capacity increment is a key question of DNA thermodynamics. It certainly does not result simply from an increase in conformational freedom on dissociation of the complementary strands: this could be responsible only for a small part of the observed heat capacity effect; neither can it be caused by exposure of polar groups on breaking the hydrogen bonds between complementary bases, because the heat capacity effect of the hydration of polar groups is negative (Privalov and Makhatadze 1992; Spolar et al. 1992). Thus, the increase of DNA heat capacity upon unfolding must result from some other mechanism: this can only be hydration of the exposed apolar surfaces of bases. As discussed in “Hydration effects,” transfer of apolar groups into water results in a considerable heat capacity increment, which is explained by ordering of water around the nonpolar groups and the gradual “melting” of this ordered water upon heating, resulting in the apparent heat capacity increment.

## DNA-protein complexes

### The DNA interaction with transcription factors

**Fig. 18:**
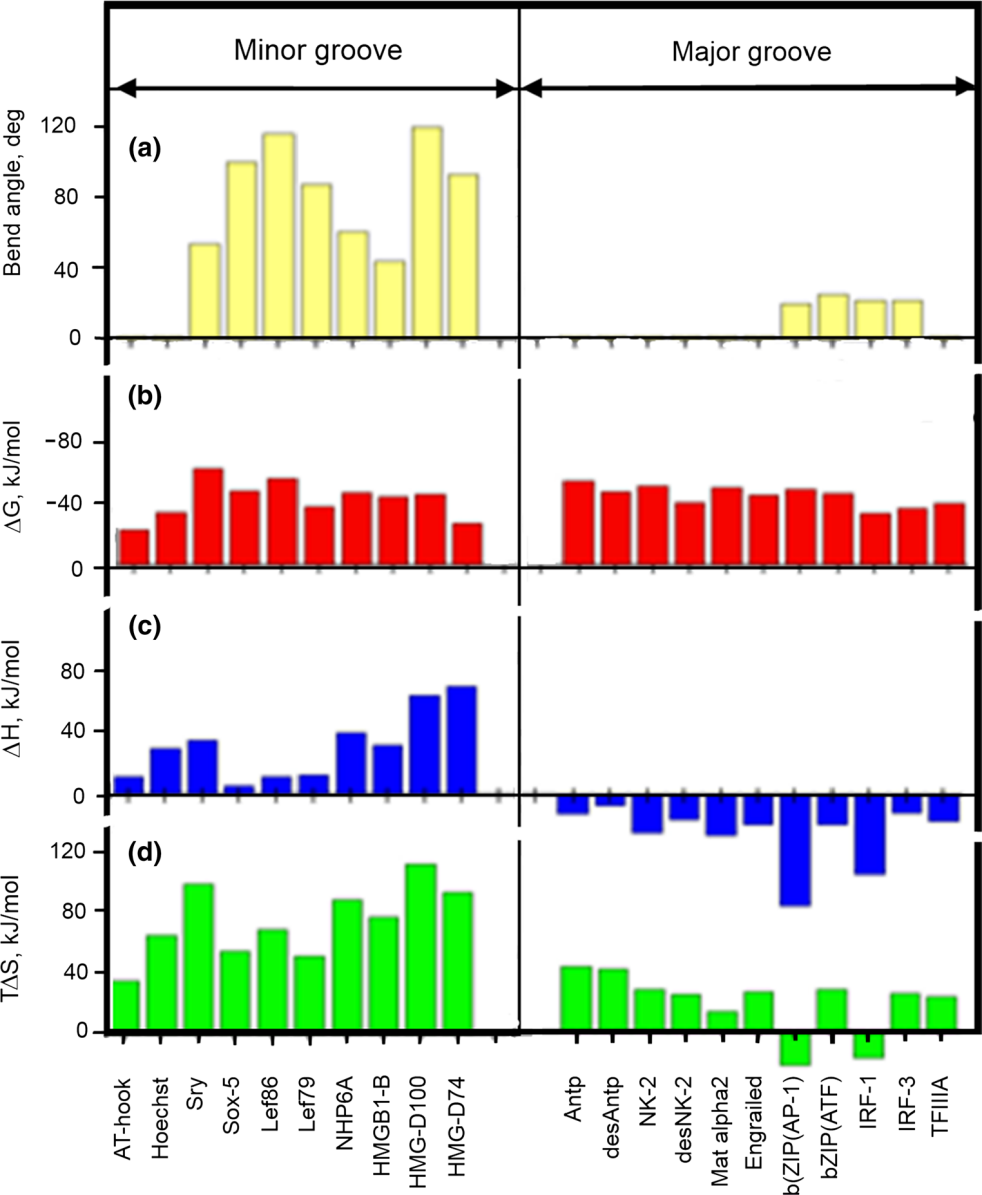
Interaction of the DBDs of various transcription factors with their target DNA sequences at 20 °C in 10 mM potassium phosphate (pH 6.0), 100 mM KCl: **a** DNA bend angles induced **b** the Gibbs energy of binding, **c** the enthalpy of binding **d**, the entropy factor of binding (for details, see Privalov et al. 2007)

Although DNA is the carrier of genetic information, the searching for required sequences and the initiation of reading this information are provided by special proteins, transcription factors, which use their DNA-binding domains (DBDs) to recognize the specific sequences. Some transcription factors bind to the major groove of DNA, while others bind to the minor groove. It is striking that binding to the minor groove usually takes place at AT-rich sequences and sometimes results in considerable DNA bending, by even more than 90 degrees (Fig. 18a).

Despite large differences in the DNA deformations caused by DBD binding, the Gibbs energies of binding to the minor and major grooves are fairly similar, around 40 kJ/mol in most cases as this provides stable enough DNA/DBD complexes at modest concentrations of the transition factors (Fig. 18b). Surprisingly, however, the enthalpies of binding to the minor and major grooves differ qualitatively: they are positive for binding to the minor groove and negative for binding to the major groove (Fig. 18c). It follows that these differences in the enthalpies are balanced by entropy factor differences (Fig. 18d).

A negative enthalpy promotes binding, while a positive enthalpy opposes it. Therefore, binding to the minor groove is driven by the entropy, which is large and positive, in contrast to the entropy of binding to the major groove, which is also positive but small in magnitude (Fig. 18c). It appears therefore that *binding of DBDs to the minor groove is entropy driven, while binding to the major grove is enthalpy driven*.

### The electrostatic and non-electrostatic components of the protein/DNA interaction

**Fig. 19:**
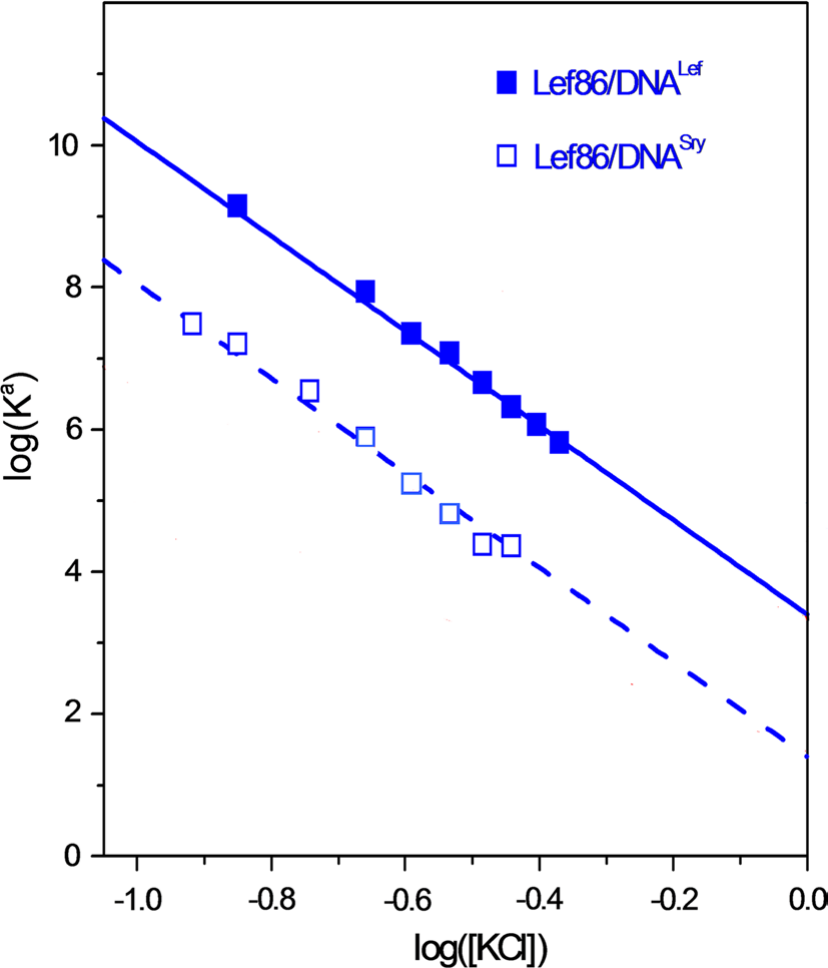
The Lef86 DBD binding to DNA^Lef^ (the optimal target) and to DNA^Sry^ (a sub-optimal sequence). The identical slopes show that the same numbers of ionic contacts are made with both target sequences. The difference in log(*K* ^a^) at log([KCl]) = 0 (and at every other KCl concentration) represents the difference in the non-electrostatic component of the interaction with the optimal and sub-optimal targets (Privalov et al. 2011)

The Gibbs energy specifying the association of various DBDs with their target DNA is not a simple parameter as it includes both enthalpic and entropic components. Moreover, although the enthalpy of DBD association with target DNA does not depend on the salt concentration, the entropy component of the Gibbs energy includes both nonelectrostatic and electrostatic terms, only the second of which depends on the salt concentration (Anderson and Record 1995; Dragan et al. 2004). The association constants show a clear logarithmic dependence (Fig. 19) expressed by the equation:

17 $$\log (K^{\text{a}} )\; = \;\log (K_{\text{nel}} ) - N \times \log [{\text{Salt}}]$$

The first term in Eq. (17) represents the non-electrostatic interactions between the protein and DNA, and the second term reflects the entropy of mixing the counterions displaced from the DNA by the bound protein with those in free solution (Manning 1978; Record et al. 1978). Extrapolating the log(*K* ^a^) function to log[Salt] = 0, where the second term vanishes, gives the non-electrostatic component of the Gibbs energy of association, ∆*G*_nel_ = −2.3 RT log(*K*_nel_). The electrostatic component of the Gibbs energy of association is then obtained as the difference from the total, ∆*G* ^a^, at the ionic strength of interest: ∆*G*_el_ = ∆*G* ^a^ − ∆*G*_nel_. Here ∆*G*_el_ is equivalent to −*T*∆*S*_el_, since the enthalpy of electrostatic interactions is zero (Anderson and Record 1995). The non-electrostatic association entropy factor is then obtained from the relation:

18 $$T\Delta S_{\text{nel}} = T\Delta S^{\text{a}} - T\Delta S_{\text{el}} .$$

**Fig. 20:**
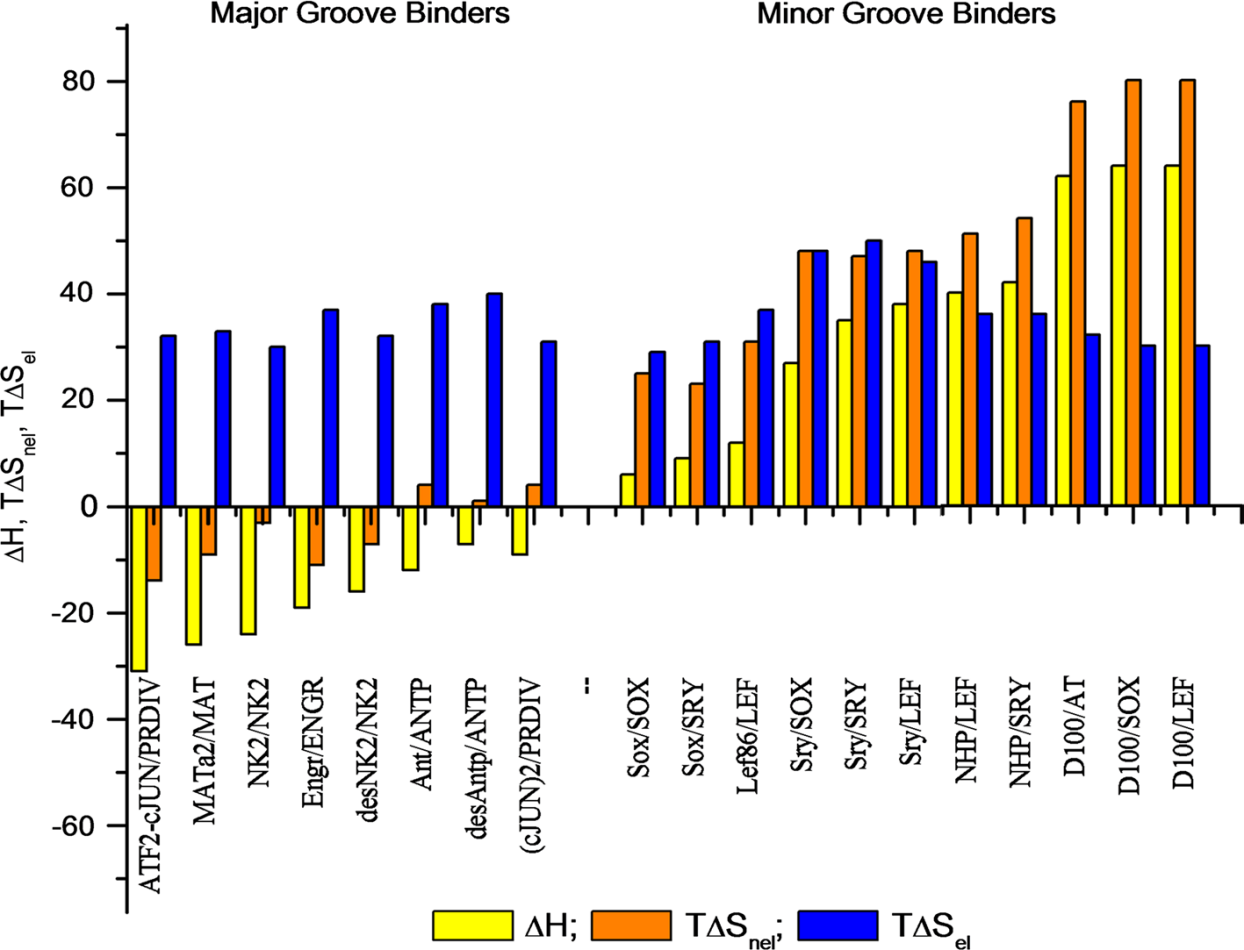
Enthalpies and entropy factors (non-electrostatic and electrostatic) of binding proteins to the minor and major groove of their optimal and sub-optimal DNAs at 20 °C in 10 mM potassium phosphate (pH 6.0), 100 mM KCl. For details, see Privalov et al. (2007)

The non-electrostatic component of the binding entropy is the sum of changes in the conformational and translational freedom of the components of the binding reaction, plus the entropy of their dehydration, while the electrostatic component derives from the release of bound counterions into the bulk solution. Figure 20 shows that the salt-dependent electrostatic component of the entropy factor, *T*∆*S*_el_ (in blue), is positive and fairly similar for all the considered DNA-protein complexes. It is a major component driving formation of the DNA-protein complexes, but *it is a non*-*sequence-specific binding force*. Sequence recognition, i.e., the specificity of binding, is provided by the binding enthalpy, ∆*H* (in yellow), plus the non-electrostatic component of the binding entropy, *T*∆*S*_nel_ (in orange), which, being salt-independent, is non-electrostatic by definition.

Considering Fig. 20, one can notice a clear correlation between the non-electrostatic enthalpy (∆*H*) and the non-electrostatic entropy factor (*T*∆*S*_nel_): *for DBD binding to the major groove both these components are generally negative, but the enthalpy substantially exceeds the entropy factor.* This rather small negative entropy factor component results mainly from the decrease in conformational and translational freedom of the DBDs and the DNA on association. *In the case of DBD binding to the minor groove the situation is drastically different.* Here both the enthalpy and non-electrostatic entropy factor are positive, and the entropy factor dominates the enthalpy. This immediately raises the question: from where do these large positive enthalpies come when DBDs bind to the minor groove of DNA?

It is notable that all DBDs that bind to the minor groove of DNA prefer AT-rich sequences, i.e., a minor groove that accommodates ordered water. It appears that *the* *removal of this ordered water upon protein binding to the minor groove gives rise to the large positive enthalpy and the especially large positive entropy of protein binding*. Dehydration of the DNA is therefore critical for protein binding to the minor groove.

### Rigidity of the DNA double helix

DNA free in solution has been characterized by the worm-like chain model as an elastic rod with a persistence length, *L*_p_, of ~50–60 nm (~150 bp) (Bustamante et al. 1994). The DNA double helix is therefore rather rigid and should not bend much on binding DBDs. This predicts substantial free energy expenditure in bending the duplex. So, how then could large bend angles of the order of 90° be generated by binding a small DBD? The most intriguing observation was that these large bends occur on binding DBDs to the minor groove of DNA at AT-rich sequences: for example, TBP (TATA box binding protein), IHF (integration host factor) and the HMG box proteins. The last are characterized by an unfavorably positive enthalpy but a favorable positive entropy, so the driving force for their binding to DNA and its bending appears to be the positive entropy of binding (Jen-Jacobson et al. 2000; Privalov et al. 1999, 2009). It was initially unclear from where the positive driving entropy derives. We now know that it results from removal of ordered water from the AT-rich minor groove of DNA (Vaitiekunas et al. 2015).

It follows that minor groove binding is used when, operationally, DNA needs to be sharply bent over only a few base pairs, and the use of AT-rich DNA sequences for this purpose appears an ingenious invention in consequence of the large entropy to be gained from release of the bound water from the minor groove with a consequent loss of rigidity. In free DNA the AT base pair, although held together by fewer hydrogen bonds than the CG base pair, nevertheless provides increased rigidity by maintaining a spine of ordered water bound in the minor groove. However, removal of this ordered ice-like water on protein binding is not energetically costly, as follows from the fact that the Gibbs energy of melting ice is close to zero at physiological temperatures. *It thus appears that the functionally important deformations of DNA can be achieved with only a small expenditure of free energy*.

## Formation of macromolecular complexes

### Translation entropy

Translational entropy is understood to mean the entropy gain/loss upon appearance/disappearance of a new kinetic unit on dissociation/formation of molecular complexes. With the realization that most biochemical reactions represent the formation or dissociation of molecular complexes, determination of the translation entropy value, particularly in an aqueous environment, became of primary importance for the quantitative specification of these processes.

The view originally proposed by Gurney (1953) was that translational entropy is expressed by the cratic term, ∆*S* ^cratic^, which is just the entropy of mixing with solvent of the additional kinetic unit appearing upon complex dissociation. This cratic entropy is assumed to be independent of the solution composition and the molecular weight of the solute. For formation of a dimer in 1 M standard aqueous solution (containing 55 mol of water) ∆*S* ^cratic^ = *R*ln(1/55) = −8.02 cal/K mol = −33.3 J/K mol. This cratic entropy was widely used in classical biophysical chemistry, e.g., by Kauzmann, Tanford and others (Kauzmann 1959; Tanford 1973). However, later it became a target of severe criticism as being physically ungrounded.

In the statistical mechanics of an ideal gas, each independent kinetic unit is specified by the translational-rotational enthalpy, $\Delta H_{{{\text{tr}}\;{ + }\;{\text{rot}}}}^{\text{o}}$, and entropy, $\Delta S_{{{\text{tr}}\;{ + }\;{\text{rot}}}}^{\text{o}}$. While translational/rotational enthalpy depends only on temperature:

19 $$\Delta H_{{{\text{tr}}{+}{\text{rot}}}}^{\text{o}} = \, 6({\text{RT}}/2) \, + {\text{ PDV }} = \, 3{\text{RT }} + {\text{ RT }} = \, 4{\text{RT}},$$

the translational/rotational entropy depends on concentration, *ρ*_o_, and some structural characteristics:

20 $$\Delta S_{{{\text{tr}}{\text{+}}{\text{rot}}}}^{{\text{o}}} = {\text{ }}S_{{{\text{tr}}}}^{{\text{o}}} + {\text{ }}S_{{{\text{rt}}}}^{{\text{o}}} = {\text{ }}[2.5R - R\ln (\rho_{{\text{o}}} \Lambda^{3} ] + [1.5R{\text{ }} + {\text{ }}R\ln \pi^{{0.5}} (8\pi^{2} kT/h^{2} )^{{3.2}} \det (A)^{{1/2}} ]$$

Here, Λ = *h*/(2π*MkT*)^0.5^, with *M* being the molecular mass of the molecule, *h* is Plank’s constant, *k* is Boltzmann’s constant, and det(A) is the determinant of the inertial tensor. The first part of this equation, also called the Sackur-Tetrode equation, expresses the translational entropy, $S_{\text{tr}}^{\text{o}}$, which thus appears to depend on the mass of the molecule (through Λ) and concentration, *ρ*_o_. The second part expresses the rotational entropy, $S_{\text{rt}}^{\text{o}}$. Unlike the translational entropy, which is concentration dependent, but is indifferent to the structure of the molecule, $S_{\text{rt}}^{\text{o}}$ does not depend on concentration but depends on molecular structure through the term det(A).

Assuming the translational entropies of macromolecules in aqueous solution do not differ from those of small molecules in the gaseous phase and can be calculated using the simple Sackur-Tetrode equation, based on the statistical mechanics of gases, the translational entropy of a typical dimeric protein at 300 K and at 1 M standard concentration gave a value of 180–230 J/K mol depending on the molecular weight of the protein (Finkelstein and Janin 1989). According to the same authors, the rotational entropy is of the same order of magnitude. Therefore, the full value of translation entropy (∆*S* ^trans^ + ∆*S* ^rot^) amounts to about 400 J/K mol, with a positive sign for the dissociation of a dimer and a negative sign for its association.

Very similar values of the entropy effects of dimerization were obtained in Tidor and Karplus (1993) using the statistical-thermodynamic approach suggested in Chandler and Pratt (1976). They calculated that dimerization of insulin results in a decrease of the translational entropy by 180 J/K mol and a decrease of rotational entropy by 200 J/K mol, but found that it should be accompanied by an increase of the vibration entropy by 110 J/K mol. Therefore, according to these authors the overall change of entropy upon dimerization of insulin should amount to −270 J/K mol.

*Thus, values of the translation entropy obtained by statistical thermodynamic analysis exceed the cratic entropy value by a whole order of magnitude.*

### Experimental verification of the translational entropy

There have been many attempts to verify the predicted translational entropy values experimentally, especially in an aqueous environment. However, experimental determination of the translational entropy in aqueous solution is not simple because it is only a part of the overall entropy of an association reaction, which also includes the entropy of dehydration of the groups removed from water upon complex formation and the entropy of conformational changes in both partners upon association. In the case of macromolecular binding reactions, these two effects substantially exceed the translational entropy effect. Therefore, the only way to determine the translation entropy on formation of a macromolecular complex appeared to be by comparing the entropy of unfolding/dissociation of the complex with the entropy of its unfolding without dissociation, i.e., unfolding of the same complex having covalently linked subunits.

There are two practical problems in the realization of such an experiment. First, the calorimetric instrument must be precise enough to reliably register the small differences between the large unfolding entropies of the two species, i.e., when covalently bound and when not. Second, the species studied should differ by only a single covalent crosslink, which holds the subunits together without any deformation, and third, the temperature-induced unfolding of these species should be highly reversible so as to be able to treat it thermodynamically.

**Fig. 21:**
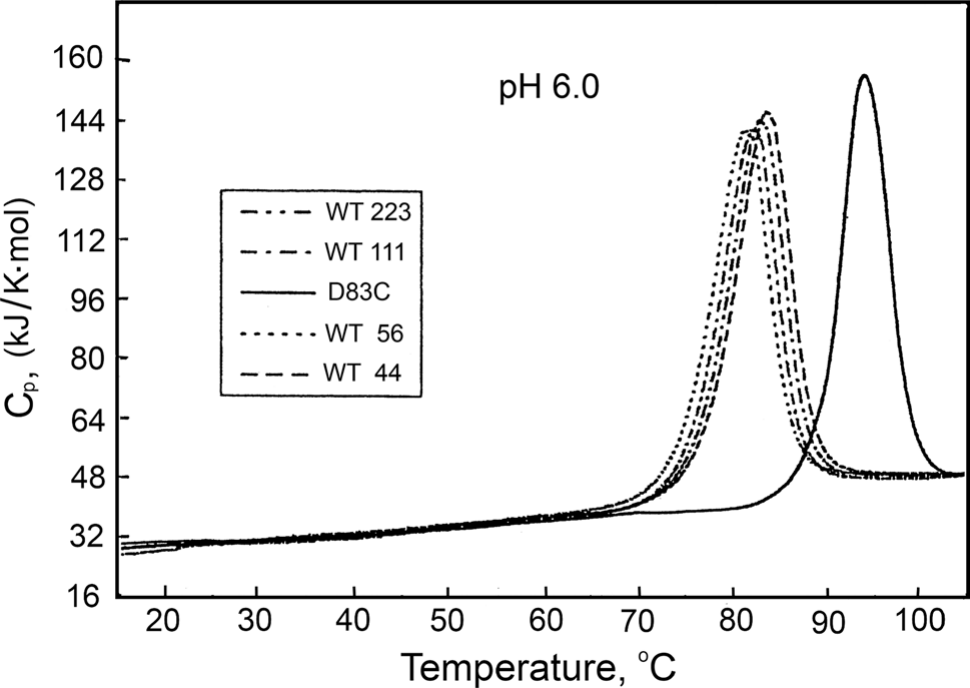
Partial molar heat capacity functions of WT SSI and the D83C mutant at different concentrations of protein in pH 6.0 solutions. *Numbers* in the *box* indicate concentrations of dimer in μM (Tamura and Privalov 1997)

The first requirement has been met by the appearance of the sensitive Nano-DSC scanning microcalorimeter having a highly stable baseline (Privalov 2012). Using this instrument, the temperature-induced unfolding was observed of the dimeric globular protein *Streptomyces* subtilisin inhibitor (WT SSI) and its mutant (D83C) with Asp83 replaced by Cys, enabling crosslinking with a disulfide bond. Using NMR and optical methods, it was shown that crosslinking does not induce noticeable changes in the conformation of SSI (Tamura and Privalov 1997). Calorimetric studies of WT SSI and its D83C mutant at various protein concentrations in solution showed that while variations of concentration do not shift the melting profile of the crosslinked D83C mutant, they have a noticeable effect on the melting profile of the WT SSI: with a decrease of protein concentration, the heat absorption peak shifts to lower temperatures and decreases in size (Fig. 21).

Analysis of the excess heat absorption profile showed that in the case of the D83C mutant, it is perfectly described by a two-state monomolecular transition, while in the case of WT SSI, it is described by a bimolecular two-state transition. For a monomolecular two-state transition, the unfolding entropy can be determined from the equation:

21 $$\Delta S(T_{\text{t}} ) = \Delta H(T_{\text{t}} )/T_{\text{t}}$$

The entropy of homodimer dissociation depends on the concentration:

22 $$\Delta S^{\text{o}} (T_{\text{t}} ) = \Delta H(T_{\text{t}} )/T_{\text{t}} + \, R\ln \{ 2[N/N^{\text{st}} ]\}$$

The first term in Eq. (22) represents the entropy of the temperature-induced transition; the second term accounts for the stoichiometry of the considered reaction at the standard concentration, *N* ^st^, which is usually 1 M. Knowing the heat capacity increment of unfolding, ∆*C*_p_(*T*), one can extrapolate the entropies measured for temperature *T*_t_ to some other temperature *T*:

23 $$\Delta S(T) \, = \Delta S(T_{\text{t}} ) - \, \Delta C_{\text{p}} \times \ln (T_{\text{t}} /T)$$

This entropy extrapolation is needed because the crosslinked and non-crosslinked dimers unfold at different temperatures but their entropies must be compared at the same temperature. The difference between the unfolding entropies of the non-crosslinked and crosslinked *Streptomyces* subtilisin inhibitor amounts to (21 ± 17) J/K mol (for details, see Tamura and Privalov 1997).

Very similar results were obtained calorimetrically by studying the melting of a homo-dimeric α-helical coiled coil, the so-called leucine zipper and its mutant in which the terminal serine was replaced by cysteine, likewise enabling crosslinking (Yu et al. 2001). Thus, upon heating the non-crosslinked coiled coil dissociates into two randomly coiled polypeptides, while the S–S crosslinked dimer forms a single random coil. From these calorimetric experiments, the difference between the unfolding entropies of the non-crosslinked and crosslinked α-helical coiled coils extrapolated to 25 °C, i.e., ∆*S* ^trans^ = (40 ± 30) J/K mol (Yu et al. 2001).

Although the error in both these measurements is substantial, the average value leads to the clear conclusion that *the experimentally determined translational entropy is more than one order of magnitude smaller than the values calculated on the basis of statistical thermodynamics and is close to the cratic entropy value suggested by classical mixing theory.*

The qualitative difference between the theoretical and experimental values of the translation entropy has precipitated an avalanche of discussion. There were many attempts to find flaws in either the calorimetric experiments or the theoretical approaches (Karplus and Janin 1999; Privalov and Tamura 1999; Tidor and Karplus 1993; Yu et al. 2001). The main target of the statistical mechanics proponents was the covalent crosslinking of dimers used in the experimental studies, despite it being shown by careful optical and NMR studies that crosslinking does not affect the structure of the dimer. In this context, it is therefore of special interest to investigate the melting of DNA duplexes of different lengths because this also permits determination of the translational entropy without any crosslinking and thus avoids concerns regarding its possible effects.

Analysis of the melting profiles of the DNA duplexes (Fig. 15) shows that the enthalpic contribution of the CG base pairs, if compared at the same temperature, is very similar, i.e*., they are additive*. Surprisingly, however, the duplex thermostability increases with the increase of the number of base pairs in the duplex. This shows that the entropy of duplex unfolding is not an additive function of the number of base pairs. This might be because, in contrast to the conformational entropy of duplex unfolding-dissociation, the translational entropy does not depend on the number of base pairs.

The entropy of hetero-duplex cooperative unfolding-dissociation can be determined using Eq. (14). Extrapolating the entropy of the more thermostable duplex 15-CG (*T*_t_ = 89.5 °C = 362.7 K) down to the melting temperature of the shorter duplex 9-CG (*T*_t_ = 74.0 °C = 347.2 K), the following two equations can set up:

24 $$\Delta S(347.2)_{{9{\text{bp}}}} = \, 847{\text{ J/K}}\;{\text{mol }} = \Delta S^{\text{trans}} + 9 \times \Delta S^{\text{conf }}$$

25 $$\Delta S(347.2)_{{15{\text{bp}}}} = \, 1473{\text{ J/K}}\;{\text{mol}} - 1.95\;{\text{kJ/K}}\;{\text{mol}} \times \ln (362.7/347.2) \, = \Delta S^{\text{trans}} + \, 15 \times \Delta S^{\text{conf}}$$

where 847 and 1473 J/K mol are the entropies of the 9 and 15 bp duplexes at their characteristic melting temperatures of 347.2 and 362.7 K, respectively.

Solving these Eqs. (24) and (25), one finds that at 347.2 K = 74 °C, ∆*S* ^conf^ = 90 J/K mol-bp and ∆*S* ^trans^ = 37 J/K mol-bp. Upon extrapolation of the temperature-dependent conformational entropy to the standard temperature of 25 °C, its value drops to 70 J/K mol-bp. Since translational entropy does not depend on temperature, its value at 25 °C will be the same, 37 J/K mol, surprisingly close to the cratic entropy. It is noteworthy *that if the translational entropy were indeed as predicted by the statistical*-*thermodynamic analysis, then the dependence of the DNA duplex stability on the number of base pairs would be one order of magnitude steeper than what is observed!*

Thus, we come again to the question: why do statistical-mechanical estimates of the translational entropy differ fundamentally from the experimental estimates of this highly important parameter? The main reason appears to be the environment in which dissociation of the dimer was considered, and this was qualitatively different in the theoretical and experimental estimations of the translational entropy, being a vacuum in the first case and water in the second. It is reasonable to expect that *the presence of water efficiently dampens vibrational, rotational and translational modes following complex dissociation, thereby reducing the entropy increase on dissociation* (Amzel 1997). However, since the translational entropy has special significance in considering biological reactions, which take place in an aqueous environment, it appears that statistical mechanics should be used with great caution in explaining living processes.

## Conclusions

The considered examples show that water is not just a liquid solvent for the organic components of living systems but is also a partner responsible for the formation of specific structures by biological macromolecules and their complexes. Moreover, water determines not only the stability of these structures but also their flexibility, which is tuned for efficient functioning at temperatures specific for the given species. Reciprocally, the biological macromolecules change the properties of water: the water that is in close contact with these macromolecules is no longer liquid but somewhat structured.

It should be noted that in this review we considered water involvement in the transformations of the structures of the biological macromolecules that are not associated with redistribution of covalent bonds. However, there are many macromolecular reactions that proceed with a change of covalent bonding, and in many of them water appears as an important component. For example, disruption of the peptide bond proceeds with uptake of a water molecule and is therefore called hydrolysis. The involvement of water in the chemical reactions of biological molecules needs, however, a separate and extended review.
